# Single-cell resolution landscape of equine peripheral blood mononuclear cells reveals diverse cell types including T-bet^+^ B cells

**DOI:** 10.1186/s12915-020-00947-5

**Published:** 2021-01-22

**Authors:** Roosheel S. Patel, Joy E. Tomlinson, Thomas J. Divers, Gerlinde R. Van de Walle, Brad R. Rosenberg

**Affiliations:** 1 Department of Microbiology, Icahn School of Medicine at Mount Sinai, 1 Gustave L. Levy Place, New York, NY 10029 USA; 2 Baker Institute for Animal Health, College of Veterinary Medicine, Cornell University, Ithaca, NY 14853 USA; 3 Department of Clinical Sciences, College of Veterinary Medicine, Cornell University, Ithaca, NY 14853 USA

**Keywords:** Horse, Single-cell RNA-seq, Peripheral blood mononuclear cells, T-bet^+^ B cells

## Abstract

**Background:**

Traditional laboratory model organisms represent a small fraction of the diversity of multicellular life, and findings in any given experimental model often do not translate to other species. Immunology research in non-traditional model organisms can be advantageous or even necessary, such as when studying host-pathogen interactions. However, such research presents multiple challenges, many stemming from an incomplete understanding of potentially species-specific immune cell types, frequencies, and phenotypes. Identifying and characterizing immune cells in such organisms is frequently limited by the availability of species-reactive immunophenotyping reagents for flow cytometry, and insufficient prior knowledge of cell type-defining markers.

**Results:**

Here, we demonstrate the utility of single-cell RNA sequencing (scRNA-Seq) to characterize immune cells for which traditional experimental tools are limited. Specifically, we used scRNA-Seq to comprehensively define the cellular diversity of equine peripheral blood mononuclear cells (PBMC) from healthy horses across different breeds, ages, and sexes. We identified 30 cell type clusters partitioned into five major populations: monocytes/dendritic cells, B cells, CD3^+^PRF1^+^ lymphocytes, CD3^+^PRF1^−^ lymphocytes, and basophils. Comparative analyses revealed many cell populations analogous to human PBMC, including transcriptionally heterogeneous monocytes and distinct dendritic cell subsets (cDC1, cDC2, plasmacytoid DC). Remarkably, we found that a majority of the equine peripheral B cell compartment is comprised of T-bet^+^ B cells, an immune cell subpopulation typically associated with chronic infection and inflammation in human and mouse.

**Conclusions:**

Taken together, our results demonstrate the potential of scRNA-Seq for cellular analyses in non-traditional model organisms and form the basis for an immune cell atlas of horse peripheral blood.

**Supplementary Information:**

The online version contains supplementary material available at 10.1186/s12915-020-00947-5.

## Background

Traditional model organisms have been invaluable in uncovering fundamental biological principles but they are not without limitations. For example, findings do not always translate between species, as has been particularly well described for mouse-human translational studies [1]. Additionally, many biological phenomena relevant to human health and society involve specific animal species, such as the circulation of emerging zoonotic pathogens in animal reservoirs [2], and the health of domesticated livestock. As such, a holistic approach to biology is essential and has been increasingly recognized by the research community and public health associations, including the World Health Organization [3–6].

Studying diverse species can prove challenging due to a dearth of experimental tools available for more commonly investigated laboratory organisms. In immunology, flow cytometry is the traditional technique for defining cell subpopulations [7, 8]. However, it relies on a priori knowledge of cell type-defining markers and highly specific antibodies against those markers [7]. Relative to human and mouse, this knowledge and the availability of these reagents is limited for many other species.

Single-cell RNA sequencing (scRNA-Seq) offers an alternative to flow cytometry for defining cell types (and their functional states) by RNA, rather than protein, expression patterns. Recent advances in scRNA-Seq technology have enabled increased throughput and decreased cost per cell, allowing researchers to process tens of thousands of cells in a single experiment [9–11]. scRNA-Seq offers many potential advantages for work in non-traditional model organisms, including (i) it is compatible across diverse species without specialized reagents, (ii) it does not rely on a priori marker selection or reagent availability, and (iii) it can be used to identify novel markers for focused experimentation [12].

In this study, we demonstrate the potential of scRNA-Seq for discerning and discovering cell types in a non-traditional model organism, the horse. Equids are agriculturally and economically important worldwide and are animal models for non-infectious immune conditions such as arthritis, asthma, the immunology of pregnancy, allergy, and immune-mediated or autoimmune disease [13–15]. They also host multiple zoonotic diseases including Eastern equine encephalitis virus, Hendra virus, methicillin-resistant *Staphylococcus aureus* (MRSA), and *Salmonella spp.* [16], and serve as models for other infectious diseases including influenza [3] and hepacivirus [17]. The study of immunologic conditions and infectious diseases in natural hosts is essential to (i) develop tools to prevent infection of animals with zoonotic diseases, (ii) break the chain of animal-to-human transmission, (iii) understand immunologic determinants of protection, clearance, and disease that could translate to improved understanding of human correlates, and (iv) improve the health of ecologically and economically important species.

Current state-of-the-art flow cytometry protocols for immunophenotyping equine PBMC [18] are unable to resolve many immune cell subtypes at high resolution. Here, we applied scRNA-Seq to characterize equine PBMC at unprecedented cellular resolution, and generate an immune cell atlas for horse peripheral blood. We identified 30 cell type clusters comprising major CD3^+^ lymphocyte, B cell, monocyte/dendritic cell (DC), and basophil cell populations. Clusters were annotated based on gene expression signatures, revealing several immune cell subtypes not previously described in horses. Interspecies comparisons with human PBMC scRNA-Seq datasets uncovered conserved blood DC subpopulations and identified a spectrum of monocyte cell states similar to humans. Remarkably, we found that a large portion of the horse peripheral B cell compartment is comprised of T-bet^+^ B cells. Cellular analogs of this population in human and mouse are associated with chronic infections [19, 20].

## Results

### Single-cell RNA-Seq of equine PBMC resolves a diversity of immune cell types

We performed scRNA-Seq on fresh PBMC collected from 7 healthy adult horses of different breeds, ages, and sexes (Table 1). In quality assessments of scRNA-Seq data processed with standard workflows (10X Genomics Cell Ranger pipeline, EquCab3.0 reference genome with Ensembl v95 transcript annotations), we observed unexpectedly low numbers of genes detected per cell (Additional file 1: Fig. S1A). Upon inspection of sequence alignments for select genes, we frequently observed reads mapped immediately downstream of annotated transcript regions (Additional file 1: Fig. S1B). This pattern is consistent with incomplete annotation of transcript 3′ untranslated regions (UTRs; the most frequent transcript region captured by 10X Chromium 3′ scRNA-Seq [21]), which is common in non-traditional model organisms relative to mouse or human reference transcriptomes [22]. We therefore implemented an optimized data processing workflow that included the End Sequence Analysis Toolkit (ESAT) [23], along with additional modifications (Additional file 1: Fig. S2; manually annotated immunoglobulin genes, quantification strategy for genes with multiple annotations, details in “Methods”). This approach significantly increased the number of genes detected per cell (Additional file 1: Fig. S1A).

**Table 1:** Characteristics of horse study subjects

Horse	Sex	Age	Breed	Cell analyzed
Subject 1	M	6	Warmblood	4614
Subject 2	F	8	Thoroughbred	5639
Subject 3	M	7	Warmblood	5870
Subject 4	M	8	Thoroughbred	4750
Subject 5	M	8	Thoroughbred	4382
Subject 6	F	9	Quarter horse	5199
Subject 7	F	10	Warmblood	4223

**Fig. 1:**
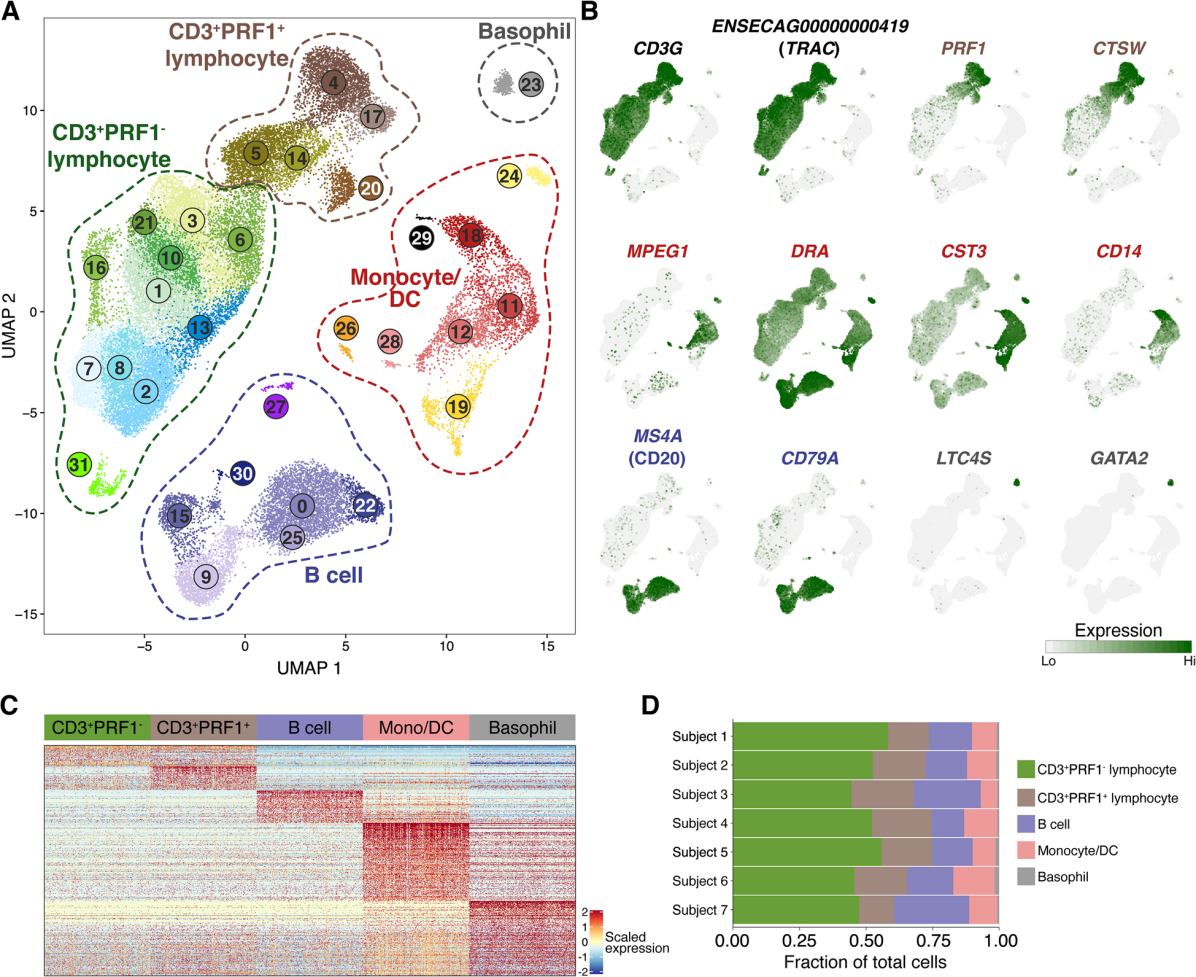
scRNA-Seq analysis of equine PBMC from 7 horses identifies five major cell groups. **a** UMAP of equine PBMC (*n* = 34,677 total cells passing filter). Points (cells) are colored by cluster membership. Dashed outlines indicate 5 major cell groups. **b** Gene expression patterns informing major cell group assignments. Expression values are scaled independently for each plot, ranging from 2.5 to 97.5 percentile of gene expression across all cells. Gene ID ENSECAG00000000419 is labeled as T Cell Receptor Alpha Chain C Region based on Ensembl/NCBI annotations. **c** Heatmap of differentially expressed genes (adjusted *p* value < 0.05, log_2_ fold-change > 1 for each major cell group versus all other major cell groups, expressed > 25% of cluster). For each major cell group, 30 cells (columns) were randomly selected from each horse for plotting purposes. **d** Frequency of each major cell group in total PBMC per horse

Unsupervised graph-based clustering of 34,677 cells integrated from the 7 horses resolved 31 clusters (Fig. 1a). Based on PCA hierarchical clustering and marker gene expression patterns (Fig. 1b, c), we grouped all clusters into 5 “major cell groups”: CD3^+^PRF1^−^ lymphocytes, CD3^+^PRF1^+^ lymphocytes, B cells, monocytes/dendritic cells (DCs), and basophils (marker gene lists in Additional file 2). All major cell groups were represented at similar proportions across all 7 horses (Fig. 1d). To characterize equine PBMC at high resolution and establish a corresponding peripheral blood immune cell atlas, we independently analyzed scRNA-Seq data for the constituent clusters of each major cell group except basophils (due to low cell counts).

### Peripheral equine myeloid cells include heterogeneous monocytes and distinct dendritic cell subsets with analogous counterparts in humans

**Fig. 2:**
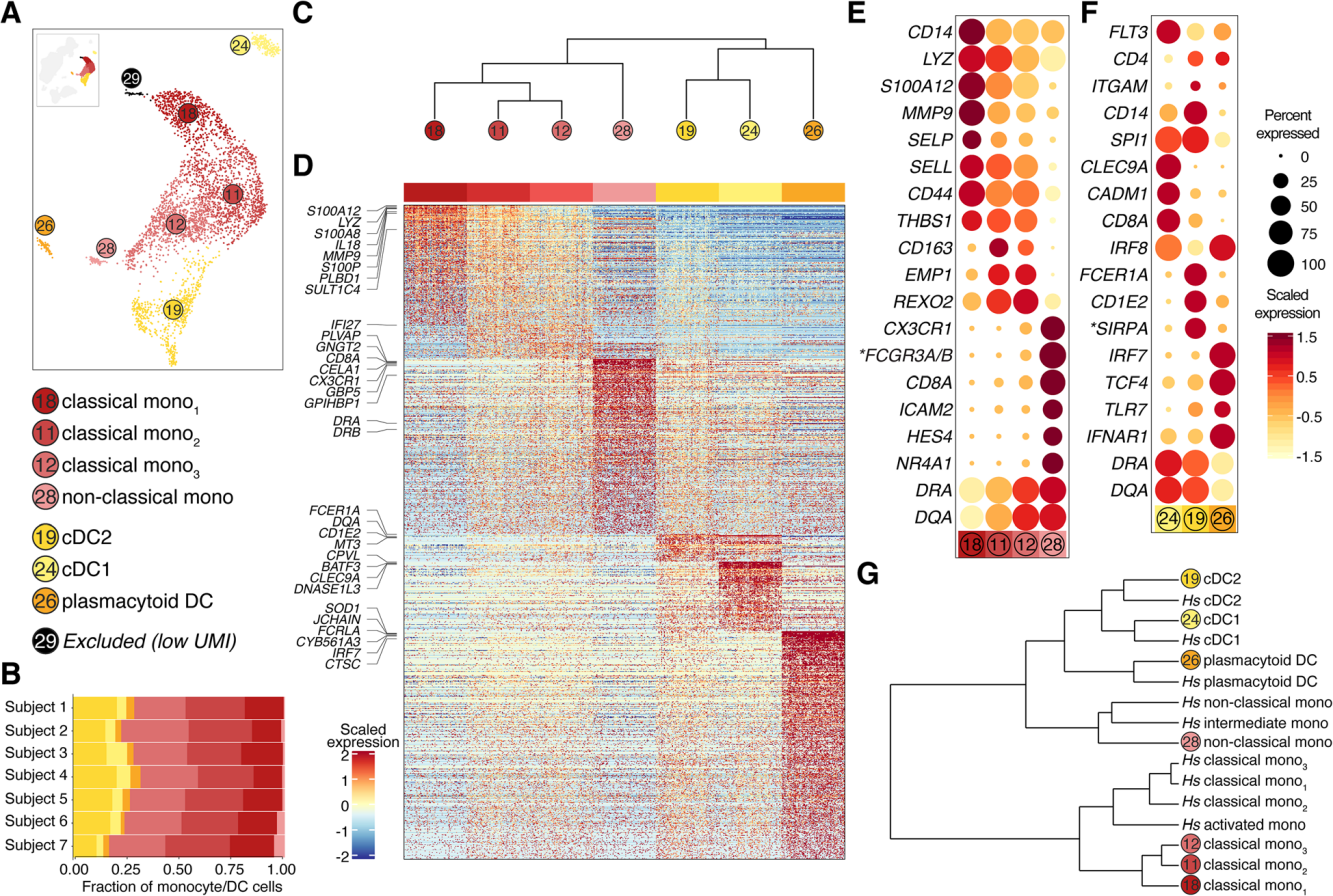
Equine monocyte/DC major cell group is comprised of diverse cell types including a range of monocyte transcriptional states and distinct dendritic cell subtypes. **a** UMAP of monocyte/DC clusters with putative cluster annotations. Cluster 29 (annotated as neutrophils) was excluded from analysis due to low transcript (UMI) counts. **b** Frequency of each cell cluster within the monocyte/DC group per horse. **c** Hierarchical clustering (integrated PCA dimensions) indicates two subpopulations, putatively annotated as monocytes (clusters 18, 11, 12, 28) and DCs (19, 24, 26). **d** Heatmap of differentially expressed genes (adjusted *p* value < 0.05, log_2_ fold-change > 1 for each cluster versus all other clusters, expressed > 25% of cluster) by cluster. **e** Dot plot of select differentially expressed genes across monocyte clusters. Dot size is proportional to number of cells with detectable expression of indicated gene. Dot color intensity indicates average gene expression values scaled across plotted clusters. *Gene ID ENSECAG00000006663 is labeled *FCGR3A/B* based on Ensembl/NCBI annotations. **f** Dot plot of select differentially expressed genes across DC clusters. *Gene ID ENSECAG00000035431 is labeled *SIRPA* based on Ensembl/NCBI annotations. Additional details as in **e**. **g** Hierarchical clustering of equine PBMC scRNA-Seq data (monocyte/DC clusters) and human PBMC scRNA-Seq data (monocyte/DC clusters). Median-normalized average expression values for highly variable human/horse one-to-one orthologs were calculated for each cluster, and clustering was performed on Pearson distances by Ward’s method

We began with a detailed characterization of the monocyte/dendritic cell clusters (Fig. 2a; clusters 11, 12, 18, 19, 24, 26, 28, and 29; cluster 29 was excluded due to low transcript (UMI) counts), which were present in similar frequencies across all horses (Fig. 2b). Hierarchical clustering on integrated PCA data suggested two distinct subpopulations (Fig. 2c). Supported by differential gene expression analysis (Additional file 3, Fig. 2d), we designated clusters 18, 11, 12, and 28 as monocytes based on expression of the canonical marker gene *CD14* [24] (Fig. 2d, e). Similarly, we designated clusters 24, 19, and 26 as presumptive DCs based on high expression of MHC II antigen presentation genes (*DRA*, *DQA*, with notably elevated relative expression in clusters 19 and 24), and significantly lower *CD14* expression (Fig. 2d, f).

Monocytes were composed of 3 abundant clusters (> 94% of total monocytes, clusters 18, 11, and 12) and 1 relatively rare cluster (< 6% of total monocytes, cluster 28). Hierarchical clustering (Fig. 2c) and heat map (Fig. 2d) visualizations suggest that clusters 18, 11, and 12 exhibit somewhat similar and/or overlapping gene expression patterns, while cluster 28 is notably transcriptionally distinct. We identified genes with significantly elevated expression in each cluster (Dataset S3). Of note, the top ranked (adjusted *p* value and log_2_ fold-change) differentially expressed gene in cluster 28 is ENSECAG00000006663, annotated as *FCGR3A/B* or *CD16*, a canonical marker for non-classical monocytes (CD14^lo^CD16^+^ by flow cytometry) in human PBMC [25]. Clusters 18, 11, and 12 demonstrate varying expression of genes associated with classical monocytes (CD14^hi^CD16^−^ in humans, Ly6C^hi^CD44^+^ in mice) and/or intermediate monocytes (CD14^++^CD16^+^ in humans), including *CD14*, *CD44*, *SELL*, and the MHC II components *DRA* and *DQA* (Fig. 2e). Additional genes with significantly elevated expression levels in cluster 28 include *NR4A1* (transcription factor necessary for differentiation of non-classical monocytes in mice) [26], *CX3CR1* (chemokine receptor characteristic of non-classical monocytes in humans and mice) [27, 28], and *HES4* (target of NOTCH signaling implicated in non-classical monocyte generation) [29] (Fig. 2e).

Presumptive DC clusters (24, 19, 26) were also analyzed by differential gene expression analysis (Additional file 5). Differentially expressed genes in cluster 24 included *CLEC9A*, *CADM1*, and *BTLA* (Fig. 2f, Additional file 5), all of which are immunophenotyping markers for cDC1 in humans and mice [30] (in mice, *CLEC9A* is also expressed on plasmacytoid DC [31]). Genes with significantly enriched expression in cluster 19 included *FCER1A* and *SIRPA* (Fig. 2f, Additional file 5), which are flow cytometric markers of cDC2 in humans and mice (Reviewed in [30]). DC subsets are also defined by the transcription factors that regulate their development and function, particularly by relative levels of IRF4 and IRF8 [30]. Although *IRF4* transcripts were sparsely detected across all DC clusters (likely due to the incomplete sampling depth characteristic of droplet scRNA-Seq), *IRF8* was expressed at high levels in cluster 24 (cDC1) and significantly lower levels in cluster 19 (cDC2). Cluster 24 also exhibited high expression of *BATF3*, another characteristic transcription factor of cDC1 [32]. In addition, top ranked differentially expressed genes in cluster 26 included *IRF7* and *TCF4* (E2–2) (Fig. 2f, Additional file 5), both of which are fundamental to plasmacytoid DC (pDC) development and function [33, 34].

To further support our cell type annotations and assess potential differences in monocyte/DC subsets between horses and humans, we performed cross-species hierarchical clustering with a human PBMC public reference scRNA-Seq data set (Additional file 1: Fig. S3A-B, Fig. 2g). Equine clusters annotated as classical monocytes clustered first with each other and next with human classical monocytes (defined by scRNA-Seq gene expression and confirmed with corresponding CD14/CD16 immunophenotyping feature barcoding data). Equine non-classical monocytes clustered with human intermediate and non-classical monocytes. Interestingly, each DC subgroup clustered by cell type rather than species, indicating strong similarities of gene expression patterns between horse and human. Taken together, these results suggest that equine monocyte populations are analogous to those described in humans and mice. Furthermore, they support three distinct DC subpopulations in horse peripheral blood that correspond with cDC1 (cluster 24), cDC2 (cluster 19), and pDC (cluster 26) in these species.

### The equine peripheral B cell compartment includes a large proportion of T-bet^+^ B cells

**Fig. 3:**
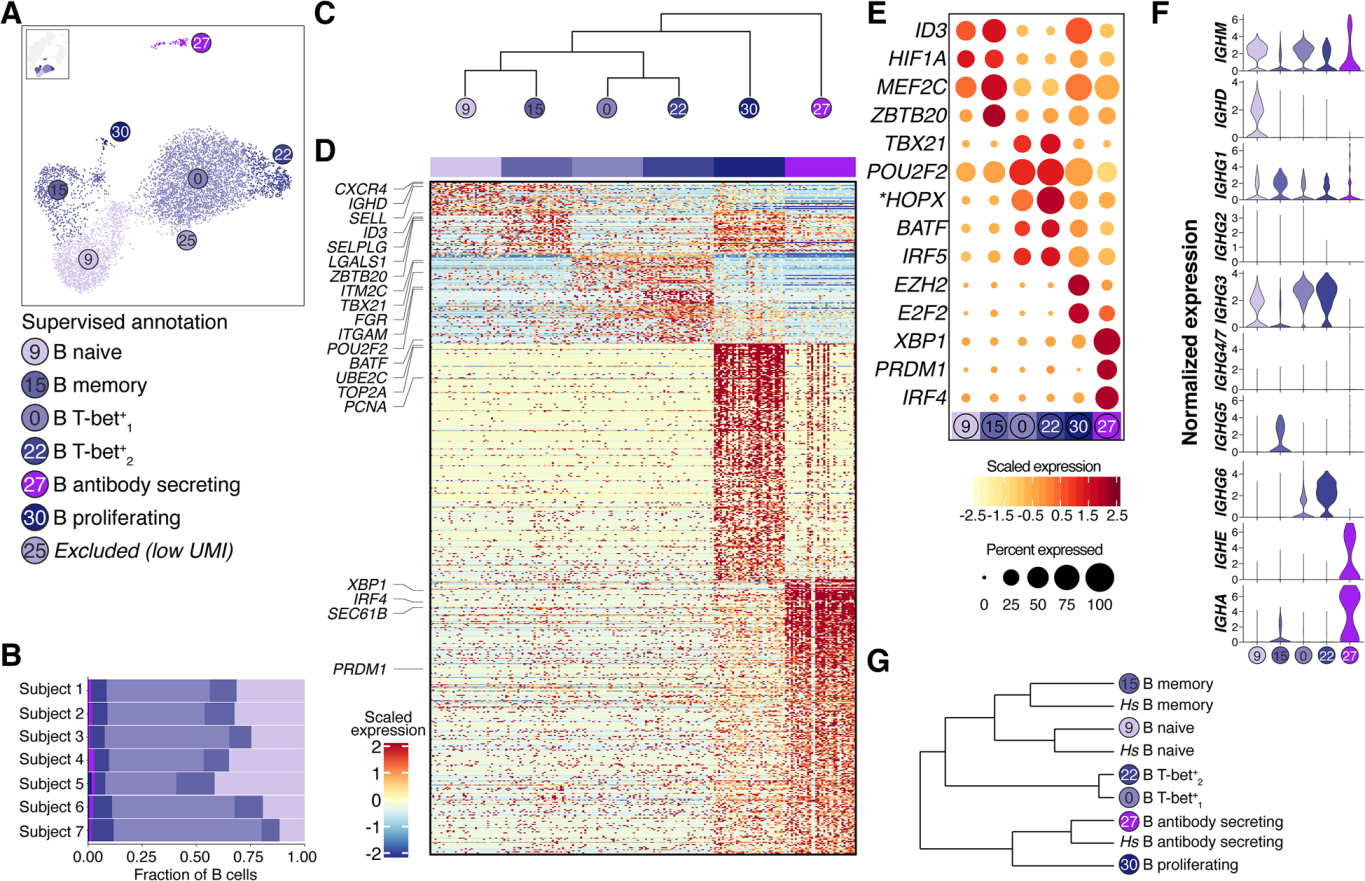
Equine peripheral B cell clusters include several distinct transcriptional states marked by expression of different transcription factors, including T-bet^+^ B cells. Clusters within the B cell major cell group were further analyzed and annotated by differential gene expression. Cluster 25 was excluded from analysis due to low transcript counts. **a** UMAP subset of B cell clusters with putative cluster annotations. **b** Frequency of each cell cluster within the B cell group per horse. **c** Hierarchical clustering (integrated PCA dimensions) of B cell major cell group. **d** Heatmap of differentially expressed genes (adjusted *p* value < 0.05, log_2_ fold-change > 1 for each cluster versus all other clusters, expressed > 25% of cluster). **e** Dot plot of select transcription factor genes differentially expressed across B cell clusters. Dot size is proportional to number of cells with detectable expression of indicated gene. Dot color intensity indicates average gene expression values scaled across plotted clusters. *Gene ID ENSECAG00000029287 is labeled *HOPX* based on Ensembl/NCBI annotation. **f** Violin plot of immunoglobulin heavychain isotype transcript expression for indicated B cell clusters. Expression values are log-normalized per cell. **g** Hierarchical clustering of equine PBMC scRNA-Seq data (B cell clusters) and human PBMC scRNA-Seq data (B cell clusters). Median-normalized average expression values for highly variable human/horse one-to-one orthologs were calculated for each cluster, and clustering was performed on Pearson distances by Ward’s method

We next performed an in-depth analysis of B cell clusters, as defined by their expression of *MS4A1* (CD20), *CD79A*, MHC II components (i.e., *DRA*), and/or immunoglobulin transcripts (Figs. 1a and 3a; clusters 9, 15, 0, 22, 25, 27, and 30; cluster 25 was excluded due to low transcript (UMI) counts, Additional file 1: Fig. S4A). We observed six B cell clusters across all seven horses (Fig. 3b); this heterogeneity was somewhat surprising given our observation of only three B cell clusters (naïve, memory, and antibody secreting) in human PBMC scRNA-Seq data (Additional file 1: Fig. S3A-B and additional datasets, *data not shown*). Hierarchical clustering on integrated PCA data suggested that clusters 27 and 30 were notably dissimilar from other B cell clusters (Fig. 3c). We annotated cluster 27 as antibody-secreting cells (ASCs; expressing *PRDM1*/BLIMP-1, *XBP1*, *IRF4*, high levels of immunoglobulin transcripts), and cluster 30 as proliferating B cells (numerous G2/M associated genes including *PCNA*, *TOP2A*, and *UBE2C*) (Fig. 3c–e, Additional file 6). Of note, ASCs, which consistently exhibited high expression of a single immunoglobulin isotype per cell, demonstrated different isotype frequencies in different horses, perhaps indicative of distinct subclinical immune challenges (Additional file 1: Fig. S4B).

For the remaining B cell clusters, we noted the restricted expression of several transcription factors associated with immune function (Fig. 3e). Consistent with hierarchical clustering (Fig. 3c), these results further suggest that B cells in clusters 9 and 15 (expressing transcription factors *ID3*, *HIF1A* and *MEF2C*) employ a different gene regulatory program than B cells in clusters 0 and 22 (defined by specific expression of *TBX21*/T-bet, as well as elevated expression of *POU2F2/*OCT-2) (Fig. 3e). Based on specific expression of *IGHD* transcripts and expression of *IGHM* transcripts, we annotated cluster 9 as naïve B cells (Fig. 3f). Relatedly, we annotated cluster 15 as likely memory B cells based on a similar gene expression signature to naïve B cells (cluster 9), but with the expression of class-switched isotype transcripts (*IGHG1*, *IGHG3*, *IGHG5*, *IGHA*), and the absence of *IGHD* transcripts (Fig. 3f). These cells are also defined by expression of *ZBTB20*, a transcription factor associated with antigen-experienced B cells (isotype-switched memory, germinal center, plasma cells) in mice [35], but they lack appreciable expression of plasma cell transcription factors such as *PRDM1*/BLIMP-1 and *XBP1* (Fig. 3e). The *TBX21*/T-bet^+^ B cells in clusters 0 and 22 exhibited diverse isotype transcript expression patterns, which included both *IGHM* and class-switched isotypes (*IGHG1*, *IGHG3*, and *IGHG6*, most pronounced in cluster 22). With sequence data restricted to 3′ transcript regions (i.e., without coverage of variable region/constant region exon-exon junction), it was not possible to infer how these RNA expression patterns relate to functional/productive immunoglobulin protein expression.

In cross-species hierarchical clustering for equine and human B cells, naïve, memory, and ASCs clustered by cell type before species (Fig. 3g, Additional file 1: Fig. S3A-B). However, the equine T-bet^+^ B cells (clusters 0 and 22) appeared on a distinct branch of the clustering dendrogram. These results support our annotation of equine naïve and memory B cell populations and suggest that the T-bet^+^ B cell clusters, which include the most abundant B cell cluster in horse peripheral blood, do not have a corresponding B cell population in PBMC from healthy humans (*N* = 2).

### Equine T-bet^+^ B cells share gene expression features with human T-bet^+^ B cells and can be identified in equine PBMC by flow cytometry

**Fig. 4:**
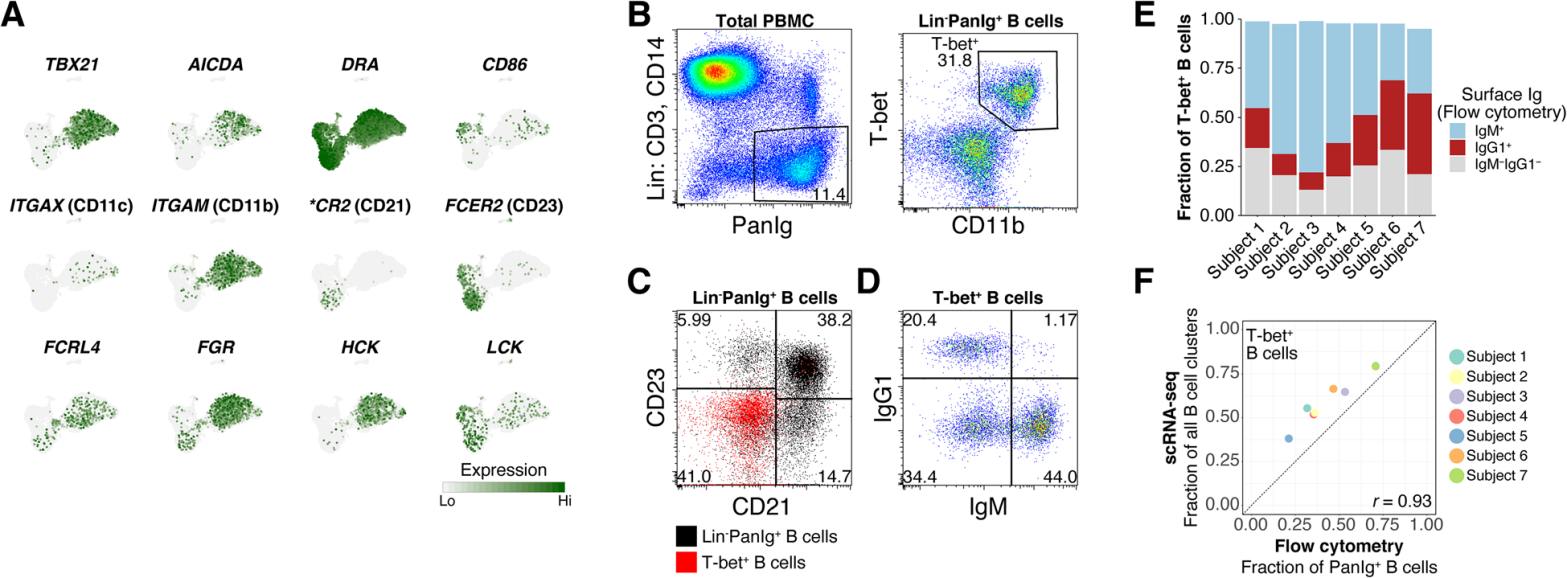
Equine T-bet^+^ B cells exhibit gene expression and cell surface protein marker expression characteristic of human T-bet^+^ B cells and express diverse surface immunoglobulin isotypes. **a** Expression patterns for select genes associated with T-bet^+^ atypical B cells in humans and/or mice. Expression values are scaled independently for each plot, ranging from 2.5 to 97.5 percentile of gene expression across all B cell clusters. *Gene ID ENSECAG00000031055 labeled as *CR2* (CD21) based on Ensembl/NCBI annotation. **b** Flow cytometry gating strategy and expression of surface markers (PanIg and CD11b) and T-bet. **c** Expression of surface markers CD21 and CD23 in T-bet^+^ B cells. **d** Expression of surface immunoglobulin isotypes (IgM, IgG1) in T-bet^+^ B cells. **e** Quantification of surface immunoglobulin isotype expression by flow cytometry. **f** Correlation of T-bet^+^ B cells as determined by scRNA-Seq compared to flow cytometry. Pearson correlation coefficient, *r* = 0.93

In humans, T-bet^+^ B cells have been described as “atypical memory B cells,” appearing in the peripheral blood during chronic infection and/or inflammation [20, 36]. Although specific markers and/or gene expression patterns vary in different datasets, these cells are often found to express *ITGAM* (CD11b) and *ITGAX* (CD11c), genes that modulate BCR signaling (including *FCRL4*, *FGR*, and *HCK*) [37–41], and genes associated with germinal center B cells such as *AICDA* (encoding activation-induced cytidine deaminase) and *APEX1* [19, 42]. We assessed expression of several of these characteristic genes, and observed patterns consistent with multiple reports in humans (Fig. 4a). Among B cells, *ITGAM* (CD11b) expression was restricted to clusters 0 and 22. Although sampling for *ITGAX* (CD11c) and *ENSECAG00000031055* (annotated as *CR2*/CD21) was insufficient for differential expression testing, we detected *ITGAX* (CD11c)-positive cells in T-bet^+^ clusters 0 and 22 (Fig. 4a). Moreover, we detected significantly elevated expression of *FCLR4*, *FGR*, and *HCK* in these clusters (Fig. 4a, Additional file 6).

We next confirmed T-bet protein expression in these cells by flow cytometry. Within CD3^−^CD14^−^PanIg^+^ B cells (Additional file 1: Fig. S5), we detected an abundant CD11b^+^ T-bet^+^ population (Fig. 4b) that did not express surface CD21 (*CR2*) or CD23 (*FCER2*) (Fig. 4c). T-bet and CD11b expression were not detected in other B cell gates. We also assessed surface isotype usage of T-bet^+^ B cells by flow cytometry; 51 ± 18% T-bet^+^ B cells were IgM^hi^, 23 ± 12% were IgG1^+^, and 24 ± 8% expressed neither IgG1 nor IgM (Fig. 4d, e). It is unclear whether IgM^hi^ T-bet^+^ B cells reflect an antigen-inexperienced naïve subset, a recently activated subset, or an IgM^+^ memory cell subset. By flow cytometry, T-bet^+^ B cells comprised 44 ± 17% of total B cells; these frequencies were correlated with, but were consistently lower than frequencies from scRNA-Seq data (Fig. 4f). These results validate the existence of a novel population of T-bet^+^ B cells initially identified by scRNA-Seq analysis, which shares similarities with human T-bet^+^ “atypical memory B cells.”

### CD3^+^PRF1^+^ clusters include lymphocytes with diverse cytotoxic gene expression patterns

**Fig. 5:**
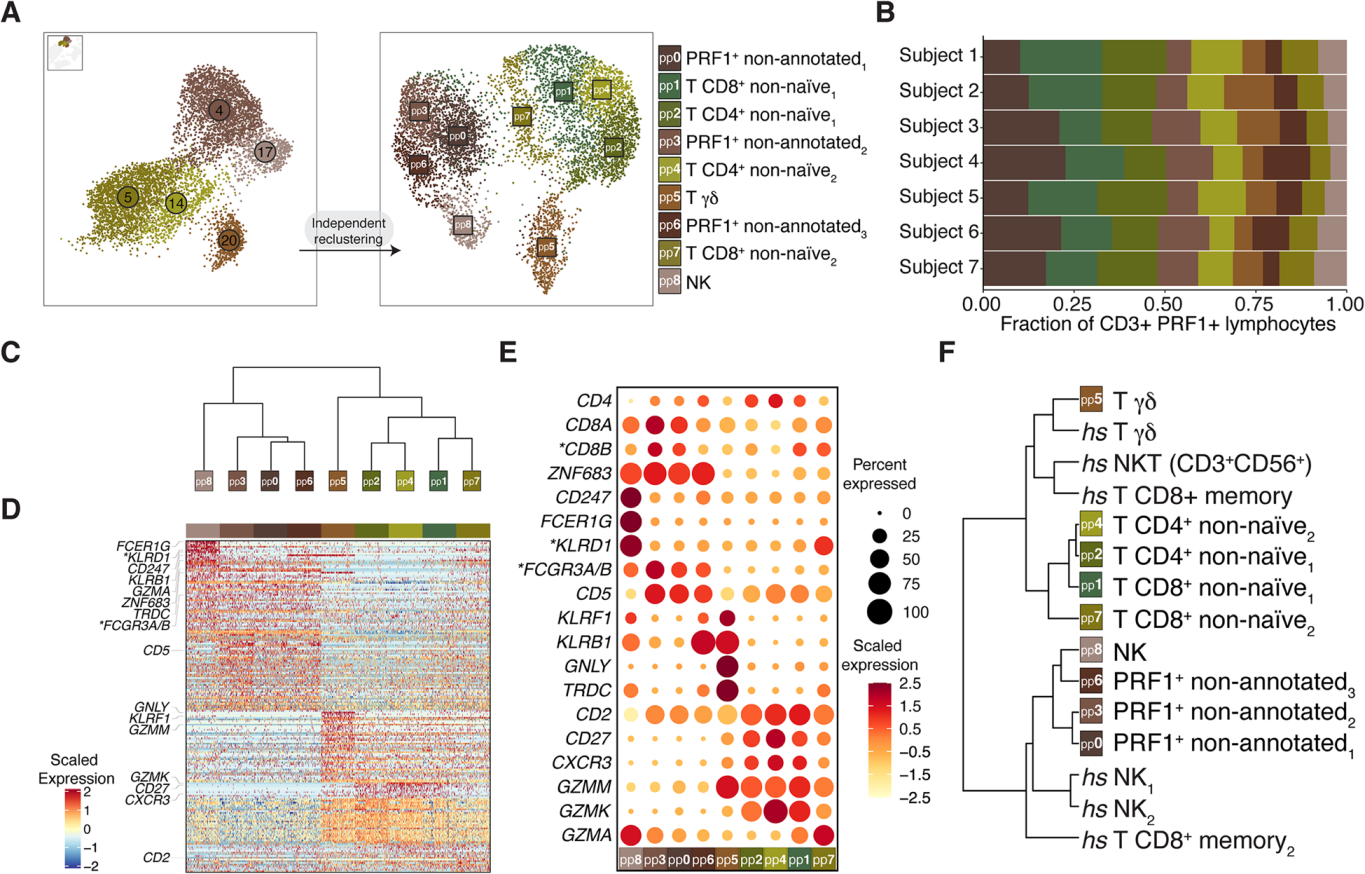
The CD3^+^PRF1^+^ clusters contain various cell types with different gene expression patterns characteristic of cytotoxic lymphocyte function. Clusters within the CD3^+^PRF1^+^ lymphocyte major cell group was further analyzed and annotated by differential gene expression. **a** UMAP subset of CD3^+^PRF1^+^ lymphocyte clusters (left) and of re-clustering analysis with putative cluster annotations (right). Selected axis ranges excluded < 5 cells in CD3^+^PRF1^+^ group from UMAP subset plot. **b** Frequency of each cell cluster within the CD3^+^PRF1^+^ lymphocyte group per horse. **c** Hierarchical clustering (integrated PCA dimensions) of CD3^+^PRF1^+^ lymphocyte major cell group. **d** Heatmap of differentially expressed genes (adjusted *p* value < 0.05, log_2_ fold-change > 0.58 for each cluster versus all other clusters, expressed > 25% of cluster). **e** Dot plot of select genes associated with cytotoxic lymphocyte populations differentially expressed across CD3^+^PRF1^+^ lymphocyte clusters. Dot size is proportional to number of cells with detectable expression of indicated gene. Dot color intensity indicates average gene expression values scaled across plotted clusters. *Gene ID ENSECAG00000006663 is labeled *FCGR3A/B* and Gene ID ENSECAG00000031528 is labeled *KLRD1* (CD94) based on Ensembl/NCBI annotations. **f** Hierarchical clustering of equine PBMC scRNA-Seq data (CD3^+^PRF1^+^ lymphocyte clusters) and human PBMC scRNA-Seq data (non-naïve CD8^+^ T cells, NK cells, NKT cells). Median-normalized average expression values for highly variable human/horse one-to-one orthologs were calculated for each cluster, and clustering was performed on Pearson distances by Ward’s method

Initial examination of CD3^+^PRF1^+^ major cell group suggested heterogeneous and overlapping cell populations. Transcriptional profiling studies of human and mouse cells often describe challenges in distinguishing cytotoxic lymphocyte subpopulations, with memory αβ CD8^+^ T cells, NK cells, NKT cells, and γδ T cells exhibiting considerable overlap in gene expression patterns [43–47]. Our data suggest similar overlap exists among equine cytotoxic lymphocyte subpopulations. To improve resolution of potentially distinct cell types, we extracted and re-clustered data from the CD3^+^PRF1^+^ group independently of the other major cell groups. While clustering assignments were generally consistent with the initial analysis (Additional file 1: Fig. S6A), independent re-clustering resulted in a total of 9 high-resolution clusters (Fig. 5a, designated with a PRF1 positive, “pp” prefix), which were represented at similar frequencies across all horses examined (Fig. 5b). All clusters were characterized by expression of the cytotoxic effector *PRF1* and *CTSW*, a cathepsin whose expression is associated with cytotoxic capacity [48] (Fig. 1b). Although overlap in gene expression across clusters remained apparent, hierarchical clustering partitioned the major cell group into at least three distinct transcriptional programs (Fig. 5c, d). Although all clusters expressed high levels of CD3 transcripts (*CD3D*, *CD3E*, *CD3G*, Additional file 1: Fig. S6B), based on differential gene expression (Additional file 8), *CD3*^+^*PRF1*^+^ cells likely include both cytotoxic T cells and NK cells.

We annotated clusters pp1, pp2, pp4, and pp7 as “antigen-experienced” or “non-naïve” T cells. Of note, we observed both *CD8A*^+^ (pp1, pp7) and *CD4*^+^ (pp2, pp4) clusters (Additional file 1: Fig. S6C), which appear to share common cytotoxic gene expression patterns. Clusters pp1, pp2, and pp4 exhibited features more consistent with CD8^+^ T central memory cells in humans (GZMK/GZMM protein expression, absence of GZMA protein), whereas cluster pp7 exhibited features more consistent with CD8^+^ T effector memory cells (GZMK/GZMM/GZMA protein expression) [49].

We annotated cluster pp5 as cytotoxic γδ T cells (Fig. 5a, d, e), based on significantly elevated expression of *TRDC* (TCR delta chain), and lower levels of *TRAC*, *TRBC1*, and *TRBC2* relative to other cytotoxic lymphocyte clusters (Additional file 1: Fig. S6D). Interestingly, this cluster demonstrated high and rather specific expression of several genes associated with cytotoxicity, including *GNLY* and *KLRF1*. These results support the existence of equine γδ T cells, which have not been definitively characterized. Moreover, these cells might employ unique cytolytic mechanisms compared to other equine cytotoxic lymphocytes.

The remaining *CD3*^+^*PRF1*^+^ clusters (clusters pp0, pp3, pp6, and pp8) exhibited gene expression patterns consistent with both cytotoxic T cells and NK cells. All clusters demonstrated high expression of TCR complex components, including *CD3D*, *CD3E*, *CD3G*, TCR alpha chain (*TRAC*, ENSECAG00000000419), and TCR beta chain (*TRBC1*, ENSECAG00000033316; *TRBC2*, ENSECAG00000030258) (Additional file 1: Fig. S6B, D). However, all clusters also displayed expression of genes associated with NK cell function, including *FCGR3A/B* (CD16, employed by NK cells for antibody-dependent cellular cytotoxicity), and *ZNF683* (HOBIT) (Fig. 5e). *ZNF683* is a transcription factor highly expressed by human NK cells [50], used as a marker for equine NK cells by RT-PCR [51], and described in human cytotoxic T cell subsets [52]. We annotated cluster pp8 as NK cells based on expression of ENSECAG00000031528 (annotated as *KLRD1*/*CD94*), which encodes the cell surface lectin central to NKG2 functions (Fig. 5e). This cluster also exhibited specific expression (within *CD3*^+^*PRF1*^+^ clusters) of *FCER1G* and *CD247*, both of which are important for NK cell activation signal transduction [53]. Additionally, this putative NK cell cluster exhibited diminished or absent expression of *CD2* and *CD5*, genes frequently used as T cell markers in humans [54] (Fig. 5e). Of note, multiple descriptions of equine NK cells by flow cytometry or immunohistochemistry have purposefully excluded CD3^+^ cells [55–57]. However, consistent with scRNA-Seq, our flow cytometric analysis identified a well-defined CD3^+^CD16^+^ lymphocyte population (Additional file 1: Fig. S7), which could correspond to cluster pp8. Given their expression of TCR transcripts, it remains unclear whether these cells have the capacity to respond to specific antigen presented by traditional MHC I or MHC II.

Although clusters pp0, pp3, and pp6 share gene expression patterns consistent with both cytotoxic T cells and NK cells, the absence of definitive marker genes and/or genes associated with NK cell-restricted functions made it challenging to annotate these similar clusters. Based on the overlapping gene expression programs described in cytotoxic lymphocytes in better characterized species, we suspect these clusters could include an additional type of CD8^+^ cytotoxic T cells, semi-invariant TCR cytotoxic T cells (e.g., mucosal-associated invariant T cells, NKT cells), and/or an additional type of NK cell. The latter possibility is further supported by cross-species comparison to human cytotoxic lymphocytes (Fig. 5f). Alternatively, these clusters may represent a novel type of cytotoxic lymphocyte unique to horses.

### CD3^+^PRF1^−^ clusters include naïve T cells and heterogeneous CD4^+^ T cell populations

**Fig. 6:**
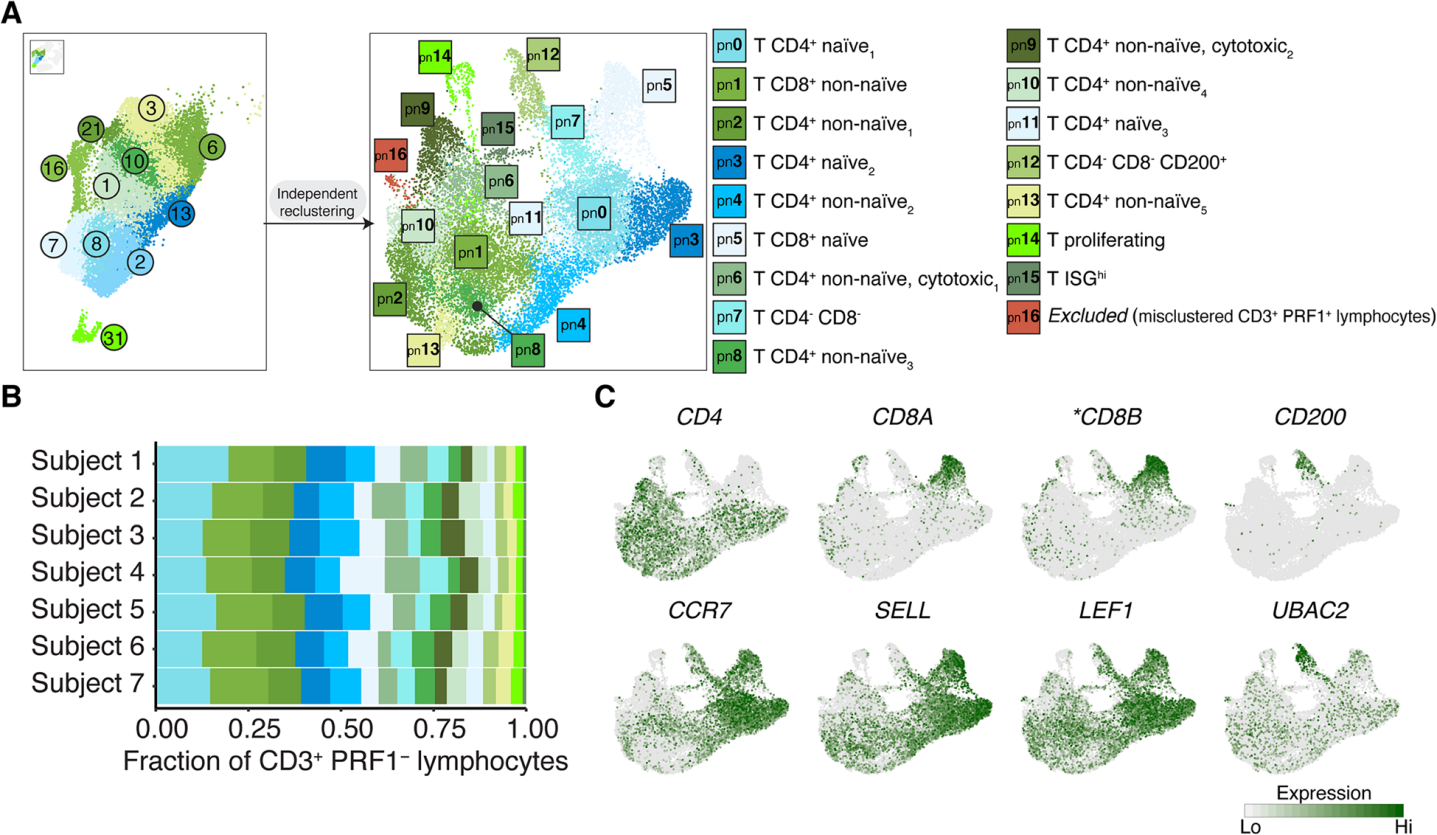
The CD3^+^PRF1^−^ clusters include naïve CD4^+^ and CD8^+^ T cells and additional CD4^+^ T cell populations. Clusters within the CD3^+^PRF1^−^ lymphocyte major cell group were further analyzed and annotated by differential gene expression. **a** UMAP subset of CD3^+^PRF1^−^ lymphocyte clusters (left) and of re-clustering analysis with putative cluster annotations (right). Selected axis ranges excluded < 10 cells in CD3^+^PRF1^−^ group from the UMAP subset plot. **b** Frequency of each cell cluster within the CD3^+^PRF1^−^ lymphocyte group per horse. **c** Expression patterns for genes characteristic of CD4/CD8 T cell subsets, naïve T cell populations (*CCR7*, *SELL*, *LEF1*), and a distinct CD3^+^CD4^−^CD8^−^CD200^+^ lymphocyte population (*CD200*, *UBA2C*). Expression values are scaled independently for each plot, ranging from 2.5 to 97.5 percentile of gene expression across all CD3^+^PRF1^−^ cells. Gene ID ENSECAG00000000775 labeled as *CD8B* based on Ensembl/NCBI annotation

As for the CD3^+^PRF1^+^ major cell group, we performed independent re-clustering on cells in the CD3^+^PRF1^−^ group. While generally consistent with initial cluster assignments (Additional file 1: Fig. S8A), re-clustering resulted in the resolution of several previously unrecognized populations, including a relatively rare group of T cells with high levels of interferon-stimulated gene (ISG) expression (Fig. 6a, Fig. S8B-C). In sum, independent re-clustering resulted in 16 clusters (Fig. 6a, designated with a PRF1 negative, “pn” prefix), which were represented at similar frequencies across all horses examined (Fig. 6b). These subpopulations were the most challenging to effectively annotate, due to the relatively subtle transcriptional differences detected between most clusters. In our experience, resting T cell populations can be difficult to distinguish by droplet microfluidics scRNA-Seq data. Despite these limitations, we were able to make several observations regarding the constituent T cell clusters. First, we distinguished naïve T cells (clusters pn0, pn3, pn5, and pn11) based on elevated expression of *CCR7*, *SELL* (L-selectin), and the *LEF1* transcription factor (Fig. 6c). Naïve T cells could be further partitioned into *CD4*^+^ (clusters pn0, pn3, pn11, not significant by differential gene expression) and *CD8*^+^ (cluster pn5) subpopulations (Fig. 6c). We also observed two clusters (clusters pn7, pn12) with minimal detectable CD4, CD8A, or CD8B expression. Of note, cluster pn12 exhibited specific expression of CD200, as well as a distinct transcriptional program that includes genes associated with cytotoxicity (NKG7, CTSW, Additional file 9); it is unclear if this cluster may represent a previously undescribed CD4^−^CD8^−^ non-naïve T cell subset in horses. We annotated cluster pn14 as proliferating T cells based on significantly elevated expression of numerous cell cycle genes (Additional file 9). Cluster pn15 (“T ISG^hi^”) showed a transcriptional program consistent with a type I and/or type II interferon response (Additional file 1: Fig. S8B-C). Despite no clinical or laboratory indications of active infection, this T ISG^hi^ population was detected in all horses (Fig. 6b).

Although most of the remaining clusters (many of which are likely antigen experienced or “non-naïve”) exhibited significant gene expression differences, we were not able to confidently assign clusters to T cell subsets traditionally defined by flow cytometry (e.g., memory Th1, memory Th2, memory Th17, regulatory T, Additional file 9).

### High-resolution landscape of equine peripheral blood mononuclear cells

**Fig. 7:**
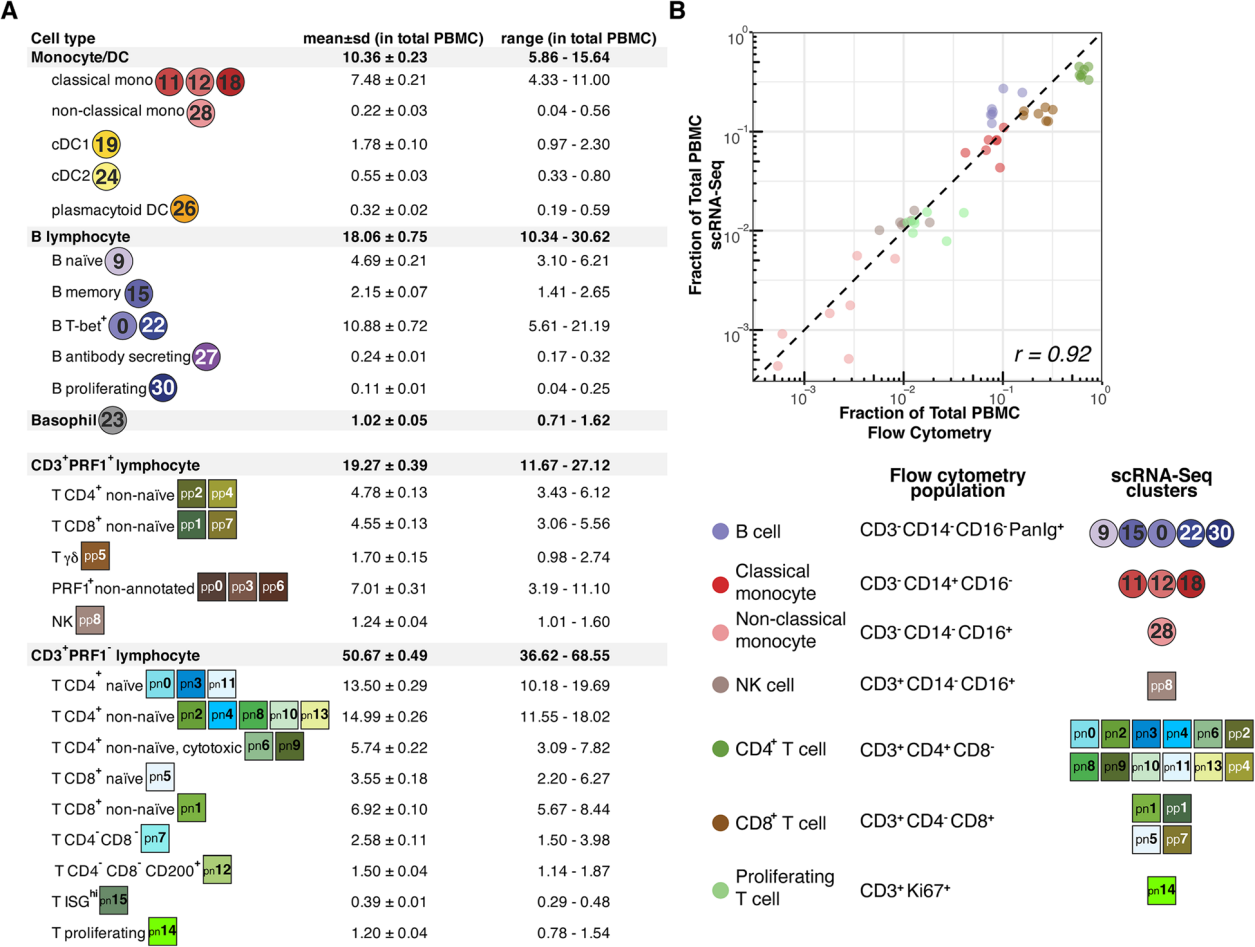
High-resolution landscape of equine peripheral blood mononuclear cells. **a** Cell type frequencies determined by scRNA-Seq. Annotated clusters were grouped to summary populations as indicated. Cell type frequencies for CD3^+^PRF1^+^ and CD3^+^PRF1^-^ groups were calculated based on results of independent re-clustering analyses. **b** Correlation of population frequencies for select cell clusters defined by scRNA-Seq with corresponding populations resolved by flow cytometry. Each point indicates cell population frequency as a fraction of total PBMC from an individual subject. Cell type definitions by flow cytometry markers and scRNA-Seq cluster summaries as indicated. Pearson correlation coefficient, *r* = 0.92

Given the improved resolution and novel cell populations identified by scRNA-Seq, we grouped annotated cell clusters into summary populations and calculated “reference ranges” for their frequency in healthy horses (Fig. 7a). Furthermore, populations that can be defined by flow cytometry gating [18] (Additional file 1: Fig. S7) were compared to corresponding scRNA-Seq clusters (grouped as indicated, Fig. 7b). Cell frequencies determined by scRNA-Seq were strongly correlated with frequencies determined by flow cytometry (*r* = 0.92 for indicated populations examined; Fig. 7b). We consistently measured higher frequencies of B cells by scRNA-Seq, which suggests that current flow cytometry definitions (i.e., PanIg^+^) based on available equine-reactive antibodies might not capture these cell populations comprehensively. These results demonstrate that our scRNA-Seq cell cluster annotations are consistent with state-of-the-art flow cytometry methods, but can resolve cell populations at much higher resolution and sensitivity.

## Discussion

Here, we used scRNA-Seq to define the cellular landscape of equine peripheral blood immune cells at unprecedented resolution. Combining supervised annotation based on prior knowledge and comparative cross-species clustering, we identified multiple cell types with immunologically relevant gene expression patterns. Many of these cell types have not been previously described in horse peripheral blood. Cross-species analyses demonstrated that many equine immune cell subpopulations have corresponding populations identifiable in humans. However, we also identified immune cell populations (e.g., T-bet^+^ B cells, discussed below) absent from healthy, steady-state human peripheral blood.

Our analysis of the monocyte/DC major cell group revealed cellular heterogeneity and subpopulations consistent with other species. Monocyte subsets, which are often categorized as classical, intermediate, and non-classical (based on surface expression of CD14 and CD16 in humans [25]), have been described as generally conserved across mammalian species [58]. Indeed, our results support the existence of similar monocyte subsets in horse and human peripheral blood. These data also include potential novel surface markers (e.g., CD8A for non-classical monocytes) for improved immunophenotyping by flow cytometry, though RNA transcript levels may not necessarily correspond with surface protein expression. We also identified three dendritic cell clusters, with gene expression consistent with the cDC1, cDC2, and pDC subsets described in humans [30] and mice [59]. Previous studies of equine DCs relied on monocyte-derived DCs in vitro [60–62]. Recently, Ziegler et al. identified DCs in equine peripheral blood based on FLT3L binding as detected by flow cytometry [63]. Guided by marker expression in other species, they proposed defining three putative DC subsets as cDC1 (Flt3^+^CD4^−^CD13^+^CD14^low^CD172a^−^CADM-1^+^MHC II^high^), cDC2 (Flt3^+^CD4^−^CD13^−^CD14^low^CD172a^+^ CADM-1^low^MHC II^high^), and pDC (Flt3^+^CD4^−^CD13^−^CD14^−^CD172a^−^CADM-1^−^MHC II^low^). With the exception of CD13 (*ANPEP*, not detected in our scRNA-Seq data), RNA transcript expression for these surface markers in DC clusters was entirely consistent with these definitions. Additional experimental characterizations are necessary to definitively assign functions to these different subsets, each of which is likely to play a critical role in equine immunity.

Our analysis also revealed previously undescribed and unexpected cell populations. Within the B cell compartment, in addition to naïve, memory, and ASC clusters, we detected two B cell clusters characterized by T-bet (*TBX21*) expression. These were the most abundant B cell clusters across all seven horses under investigation. In contrast, corresponding T-bet^+^ B cells were not observed in healthy human PBMC scRNA-Seq data. In mice, T-bet^+^ B cells have been shown to be important for antiviral humoral immunity [36, 64, 65]. In humans, T-bet^+^ B cells have been detected in peripheral blood in a variety of chronic inflammatory contexts including systemic lupus erythematosus [66, 67], chronic malaria exposure [68, 69], and chronic viral infection [19, 42, 70]. Although a universal definition and function for these cells remains elusive, T-bet^+^ B cells are often classified as atypical memory B cells and, at least in some contexts, are thought to arise from repetitive BCR stimulation [20]. Equine T-bet^+^ B cells share many features with the atypical memory B cell populations described in humans, including enriched expression of genes which modulate BCR signaling [40], and genes characteristic of germinal center B cells [19]. If these cells are elicited by chronic antigenic stimulation, it is plausible that chronic exposure to numerous pathogens common in standard boarding conditions (e.g., equine alpha and gamma herpesviruses, influenza, rhinitis viruses, hepacivirus, parvovirus, coronavirus) could expand this population. Horses in the northeastern USA are also frequently exposed to *Borrelia burgdorferi* (agent of Lyme disease) [71] and *Sarcocystis neurona* (agent of equine protozoal myeloencephalitis) [72] and are continuously infested with or re-exposed to gastrointestinal nematodes [73]. The horses in this study did not show signs of active infection or inflammation, as they all had normal complete blood count, serum amyloid A, iron indices, and globulins. Moreover, the surprisingly high frequency of these T-bet^+^ B cells suggests that they might provide important functions in the sustained immune responses to such pathogens. The impact of pathogen exposure on the genesis of this B cell population might be further explored by experiments in foals and/or in pathogen-free facilities. Additionally, horses could represent a useful model organism in which to study this unique B cell population further, given their abundant frequency and ready accessibility of large amounts of blood and other tissues, such as lymph nodes.

scRNA-Seq is molecularly compatible with presumably any animal species, as most droplet microfluidics scRNA-Seq platforms select mRNAs for barcoding and downstream sequencing based on polyA tails, a feature common across metazoans. Additional requirements for scRNA-Seq analysis include a genome (or at minimum, transcriptome) sequence to which reads are mapped, and gene/transcript annotations against which mapped reads can be quantified. Should transcriptome annotations be insufficient for robust scRNA-Seq analysis, as may be the case for less commonly studied organisms, read assignment/quantification strategies can be modified with specialized software tools (e.g., ESAT [23], as implemented here) and/or annotations can be supplemented/replaced with custom annotations derived from bulk RNA-Seq data. Interpretation of scRNA-Seq results can be greatly facilitated by gene/transcript annotations with comprehensive ortholog annotations for multiple species, but this is not a requirement. Without the need for species-specific reagents, and with a constantly growing catalog of species with sequenced and annotated genomes, we anticipate that scRNA-Seq will be an increasingly useful research tool for non-traditional model organisms.

Despite the many insights gained from our PBMC analyses, scRNA-Seq is not without limitations, particularly for characterizing cell mixtures from diverse animals. In the present study, defining subpopulations with unsupervised clustering methods was reasonably straightforward, although assigning putative cell types to each cluster presented challenges. Ideally, automated cell type classification based on external datasets and/or prior knowledge could minimize biases introduced by supervised annotation [74, 75]. Recently developed scRNA-Seq data integration and cluster annotation tools have begun to implement this functionality [76–78]. We made attempts to apply several of these strategies in comparing equine PBMC to human PBMC, but observed generally poor performance, which we attributed to insufficient interspecies ortholog annotations (*data not shown*). Instead, we adopted a supervised approach based on prior knowledge of human and mouse immune cells to assign likely cell types. We therefore emphasize that our presumptive cell type annotations are not definitive and ultimately require experimental validation by complementary methods, as we pursued with flow cytometry for T-bet^+^ B cells (Fig. 4). Furthermore, for many clusters, most notably in the CD3^+^PRF1^−^ lymphocytes major cell group, we were unable to confidently assign cell types due to limited detection of informative differentially expressed genes. This could be a result of relatively low transcript sampling depth, and/or discrepancies in mRNA and corresponding protein expression by which T cell subsets have been previously defined. Many of these issues are likely to be mitigated in the future by perennially improving genome and ortholog annotations, scRNA-Seq methodologies with increased per cell sampling depth, and novel software tools for intra- and interspecies data analyses.

## Conclusions

Our study establishes a cellular atlas of equine PBMC in healthy horses across different breeds, ages, and sexes. Many of the cell populations identified have analogous counterparts in human PBMC, including monocyte and dendritic cell subsets. A majority of the equine peripheral B cell compartment is comprised of T-bet^+^ B cells, a subpopulation that has been associated with inflammation and infection in other species. Taken together, these results demonstrate proof-of-concept for characterizing complex cell populations in non-traditional model organisms by scRNA-Seq.

## Methods

### Research subjects and cells

Horses were 3 mares and 4 geldings, 6 to 10 (mean 7.9) years old, comprised of 3 Warmbloods, 3 Thoroughbreds, and one Quarter Horse. Horses were healthy by physical examination, serum biochemistry (including globulins and iron indices), complete blood count, fibrinogen (by heat precipitation method), and serum amyloid A. Samples were processed at the New York State Animal Health Diagnostic Center on automated analyzers ADVIA 2120i (Siemens Healthcare Diagnostics Inc., Tarrytown, NJ, USA) for hematology and Cobas C501 (Roche Diagnostics, Indianapolis, IN, USA) for biochemistry. Subjects 6 and 7 had mildly elevated fibrinogen (400 mg/dL, reference interval < 200 mg/dL) with all other parameters within normal limits, including serum amyloid A < 5 μg/ml (reference interval 0–8 μg/ml). Horses were maintained in stalls with partial days spent in pasture (*n* = 4) or on pasture alone (*n* = 3) and had free access to grass or grass hay. All horses received annual core vaccinations (Eastern and Western Equine Encephalitis, West Nile Virus, Tetanus and Rabies) and at least biannual deworming (products varied). Blood samples were obtained in the morning (8–9 am), at least 16 h after the last grain meal. Subject 1 was sampled in August, Subject 3 in September, and the remaining subjects in November, all in 2018.

Approximately 50 mL of blood was collected from each horse by standard jugular venipuncture. Immediately following collection, PBMC were isolated by Ficoll gradient centrifugation, as previously described [18]. Residual erythrocytes were removed by ammonium chloride lysis. All studies were conducted under approval of Cornell University Institutional Animal Care and Use Committee (#2014-0024).

### Single-cell RNA-Seq

Within 1 h of isolation, fresh PBMC were processed for scRNA-Seq on the 10X Genomics Chromium platform (10X Genomics). PBMC collection and scRNA-Seq were performed in three independent batches (Batch 1: Subject 1, Batch 2: Subject 3, Batch 3: Subjects 2, 4, 5, 6, 7). For each PBMC sample, 9000 cells were loaded to a single lane on the 10X Genomics Chromium instrument. scRNA-Seq libraries were prepared with the 10X Genomics Chromium Single Cell 3′ Reagent Kit (v2), according to the manufacturer’s instructions. Libraries were pooled and sequenced on the Illumina NextSeq 500 in paired-end configuration (Read 1, cell barcode: 26 nt; Read 2, transcript: 98 nt) to a target read depth of approximately 35,000 paired-end reads per cell.

### scRNA-Seq data processing

scRNA-Seq data are available in the NCBI GEO repository, accession number *GSE148416* [79]. Analysis R code is available on GitHub, *BradRosenbergLab/equinepbmc* [80].

#### Reference genome and transcript annotations

The EquCab3.0 reference genome [81] was used in all analyses. Reference transcript annotations (Ensembl v95) were supplemented by manual annotation of the immunoglobulin heavy-chain and light-chain loci as described by Wagner, et al. (Additional file 7, [82]).

#### Read mapping and quantification

Reads were assigned to cell barcodes, mapped, and quantified per gene using the Cell Ranger workflow (v 3.0.1, 10X Genomics) with default parameters (“standard workflow”). In our optimized workflow, BAM files generated by Cell Ranger were reformatted (appending cellular barcode and UMI sequence to alignment read names) and were input to the End Sequence Analysis Toolkit (ESAT [23];). Briefly, ESAT evaluates reads mapped immediately downstream of annotated genes for potential quantification with the adjacent gene, an approach particularly relevant to 3′ scRNA-Seq data with reference transcriptomes with incomplete 3′UTR annotations. To eliminate ambiguous read assignments due to “overlapping genes” (i.e., exons from two different genes on + and – strands sharing the same genomic coordinates), the immunoglobulin-supplemented reference transcriptome (Ensembl v95) was additionally modified to remove overlapping exon intervals on opposite strands. Reformatted Cell Ranger BAM files were processed through ESAT in two rounds. First, ESAT was run (-wExt 2500) with the modified transcriptome reference and set to ignore any duplicated genes. Next, to recover quantification of genes duplicated in the Ensembl v95 reference (*n* = 185 duplicated genes), ESAT was run (-wExt 2500) a second time with a filtered reference containing only duplicated genes; resulting read counts were divided across gene duplications and appended to the initial gene × cell count matrix.

#### Doublet removal

Putative “doublet” cell barcodes were identified and removed from downstream analyses with the DoubletDetection tool [83].

### scRNA-Seq data analysis—equine PBMC

Gene-cell count matrices processed in the above workflow were analyzed in Seurat (v3.1.0, [76, 77]) as follows.

#### Filtering, normalization, and data integration

Data were filtered to exclude genes detected in less than 3 cells (per subject), to exclude cells with less than 750 UMIs, and to exclude cells with greater than 5% UMIs assigned to mitochondrial genes (e.g., dead or dying cells). Gene-cell count matrices were independently normalized with SCTransform [84], and the top 5500 most variable genes (variance-stabilizing transformation) were selected for each subject. To minimize subject- and/or batch-specific effects, datasets from all subjects were integrated on the first 40 canonical correlation components identified on the union of highly variable genes identified per subject. Immunoglobulin heavy-chain and light-chain genes were excluded from integration and clustering analysis.

#### Unsupervised graph-based clustering

Dimensionality reduction of the integrated dataset was performed by principal component analysis (PCA). Unsupervised graph-based clustering (smart local moving algorithm [85], resolution 1.2) was performed on the first 25 principal components (selected by Scree plot visualization). Data annotated with corresponding clusters were visualized by Uniform manifold approximation and projection (UMAP; n.dims: 25, n.neighbors: 75, cosine metric, min.dist: 0.6) [86].

To better resolve putative subpopulations in the CD3^+^ lymphocyte compartments (CD3^+^PRF1^+^ and CD3^+^PRF1^−^ major cell groups), cells from these groups were independently re-clustered with a similar workflow. Per subject data from each major cell group (i.e., CD3^+^PRF1^+^ or CD3^+^PRF1^−^) were extracted and independently normalized with SCTransform [84] to define sets of highly variable genes within each group. The top 5500 most variable genes across all subjects were selected (union method) and used for integration with Seurat’s PrepSCTIntegration, FindIntegrationAnchors and IntegrateData functions (developer’s defaults). Immunoglobulin heavy-chain and light-chain genes were excluded from integration and clustering analysis. Dimensionality reduction using PCA, unsupervised graph-based clustering, and UMAP visualization were conducted using the developer’s defaults. Clustering resolution (resolution: 0.8) was chosen by evaluation of cluster stability using the clustree package [87].

#### Differential gene expression analysis

Differential gene expression analyses were conducted using *edgeR* v3.26.8 [88, 89], with additional modifications for scRNA-Seq data [90]. Gene expression linear models included factors for cellular gene detection rate, subject, and cluster (as identified in Seurat analysis above). Specific contrasts are detailed in relevant “Results” sections and/or figures. For analyses other than comparisons among CD3^+^PRF1^+^ and CD3^+^PRF1^−^ cell clusters, differential gene expression was defined as adjusted *p* value < 0.05 (Benjamini-Hochberg correction) and moderated log_2_ fold-change > 1 (as determined in edgeR model). Differential gene expression for CD3^+^PRF1^+^ and CD3^+^PRF1^−^ cell comparisons used a less stringent fold-change cutoff (moderated log_2_ fold-change > 0.58) to account for reduced dynamic range of gene expression observed in these clusters. For all analyses, genes expressed (i.e., greater than or equal to 1 UMI) in less than 25% of cells for at least one group within a contrast were excluded from differential expression results. Resulting differential gene expression lists were further annotated for putative surface protein expression by intersecting one-to-one gene orthologs with the Human Surface Protein Atlas [91].

### scRNA-Seq data analysis—human PBMC

Human PBMC scRNA-Seq datasets (pbmc_10k_v3; pbmc_10k_protein_v3) were obtained from 10X Genomics (https://support.10xgenomics.com/single-cell-gene-expression/datasets). Sample pbmc_10k_v3 included gene expression data from 7255 human PBMC processed by 10X Chromium 3′ scRNA-Seq v3 chemistry. Sample pbmc_10k_protein_v3, 10,000 cells, also processed by 10X Chromium 3′ scRNA-Seq v3 chemistry, included gene expression data and immunophenotyping feature barcoding data for the following cell surface markers: CD3, CD4, CD8a, CD14, CD15, CD16, CD56, CD19, CD25, CD45RA, CD45RO, PD-1, TIGIT, CD127, IgG2a isotype control, IgG1isotype control, IgG2b isotype control. Gene-cell count matrices (and corresponding antibody count-cell matrix for sample pbmc_10k_protein_v3) were analyzed in Seurat v3.1.0 [76, 77].

Human PBMC scRNA-Seq data were filtered using the same workflow and parameters as above for equine PBMC. Data were normalized by SCTransform [84]. The two human PBMC datasets were integrated on the first 30 components identified by CCA. Clustering (smart local moving algorithm [85], resolution 1.2) was performed on the first 35 principal components (selected by Scree plot visualization), and results were visualized by UMAP. Resulting clusters were annotated based on surface marker antibody labeling from sample pbmc_10k_protein_v3, as described in the text and associated figure legends.

### Horse-human PBMC scRNA-Seq cross-species correlation analysis

Cross-species scRNA-Seq correlation analyses were conducted using an approach based on Zilionis et al. [92]. Human and horse gene-cell count matrices were filtered to keep only those genes with high confidence 1-to-1 orthologs across species (as defined by Ensembl v95). For each species and each major cell group (monocyte/dendritic cells, B cells, CD3^+^PRF1^+^ lymphocytes, CD3^+^PRF1^−^ lymphocytes), following normalization with SCTransform [84], genes were ranked by Pearson residual, and genes above the 1.5*inflection point were selected as highly variable genes. Lists of highly variable genes in human and horse datasets were intersected, and the resulting list of orthologs present in both species was used for clustering analysis. Clustering was performed on natural log-normalized gene × cluster count matrices and clustered on Pearson correlation distance by Ward’s method [93]. Results were visualized by dendrogram with the *dend* function in *R*.

### Immunophenotyping of equine PBMC by flow cytometry

All flow cytometry data is available on Flow Repository, accession number FR-FCM-Z2JN.

The flow cytometric phenotyping protocol was adapted from [18]. A list of primary antibodies is included in Additional file 10: Table S1 [94–100]. Unconjugated primary antibodies CD23 and IgM were conjugated with Mix-n-Stain fluorescent protein tandem dyes antibody labeling kit for APC-CF750T (Biotium, Fremont, CA, USA) and Mix-n-Stain cf. dye antibody labeling kit for CF405M (Biotium, Fremont, CA, USA), respectively, according to the manufacturer’s instructions. All wash steps were 2 ml PBS, and all labeling was performed at 4 °C for live cells and room temperature (RT) for fixed cells. Panel M included antibodies against CD3-AF647, CD14-biotin, CD16-unconjugated, and PanIg-PE. Cells were blocked with 2% fetal bovine serum for 15 min and incubated with anti-CD16 for 30 min. Cells were washed, blocked with 10% goat serum for 15 min, and incubated with secondary antibody for 30 min. Cells were washed, incubated with the remaining monoclonal antibodies to surface antigens for 30 min, and washed. Streptavidin-pacific orange was applied for 30 min to label CD14-biotin. Cells were washed and resuspended in PBS with 7AAD viability stain.

Panel T included antibodies against CD3-AF647, CD14-biotin, CD21-BV421, CD4-FITC, CD8-RPE, and Ki67-PECy7. Cells were labeled with a fixable viability marker live/dead near IR for 30 min and washed, and the surface cocktail followed by streptavidin was applied as for Panel M. Cells were then fixed (eBioscience™ Intracellular fixation and permeabilization buffer set, Thermo Fisher Scientific, Waltham, MA, USA) at RT for 30 min, washed in permeabilization buffer, incubated with antibody for the intracellular marker Ki67 for 30 min, washed, and resuspended in PBS.

Panel B1 included antibodies against PanIg-PE, CD3-AF647, CD14-AF647, T-bet-PECy7, CD21-BV421, CD23-APC-CF750, and CD11b-PerCP-Vio700. Panel B2 included antibodies against PanIg-PE, CD3-AF647, CD14-AF647, T-bet-PECy7, IgM-CF405M, and IgG1-AF488. Cells were labeled with fixable viability marker live/dead aqua for 30 min and washed, and the surface cocktail was applied. Cells were then fixed (TrueNuclear™ TF fixation and permeabilization buffer set, BioLegend, San Diego, CA, USA) at RT for 60 min, washed in permeabilization buffer, incubated with the intranuclear marker T-bet for 30 min, washed, and resuspended in PBS.

Fluorescence was measured on a Gallios flow cytometer (Beckman Coulter, Indianapolis, IN, USA) with a minimum 100,000 events collected per sample. Analysis was performed with FlowJo version 10.6.1 (FlowJo LLC, Ashland, OR, USA). Single-color controls were used to set the compensation matrix. Gating strategies are shown in Additional file 1: Fig. S7. The researcher performing gating analyses (J.E.T) was blinded to scRNA-Seq results.

## Supplementary Information

**Additional file 1: Figure S1.** Optimized scRNA-seq data processing workflow improves per cell gene detection. **Figure S2.** scRNA-seq data processing workflow. **Figure S3.** Human reference scRNA-seq clustering results and annotation. **Figure S4.** B cell quality control metrics and antibody secreting cell immunoglobulin isotype usage. **Figure S5.** T-bet^+^ B cells identified by scRNA-Seq are detectable by flow cytometry in all subjects examined. **Figure S6.** Select gene expression patterns in CD3^+^PRF1^+^ lymphocyte major cell group. **Figure S7.** Representative flow cytometry gating schemes for immunophenotyping of equine PBMC. **Figure S8.** CD3^+^PRF1^−^ lymphocyte major cell group includes lymphocytes with high expression of ISGs.

**Additional file 2.** Differentially expressed genes for major cell groups.

**Additional file 3.** Differentially expressed genes for monocyte and dendritic cell clusters.

**Additional file 4.** Differentially expressed genes within monocyte clusters only.

**additional file 5.** Differentially expressed genes within dendritic cell clusters only.

**Additional file 6.** Differentially expressed genes for B cell clusters (excluded cluster 25).

**Additional file 7.** Immunoglobulin reference gene annotation file, adapted from Wagner et al. [75].

**Additional file 8.** Differentially expressed genes for CD3^+^PRF1^+^ cell clusters.

**Additional file 9.** Differentially expressed genes for CD3^+^PRF1^−^ cell clusters.

**Additional file 10: Table S1.** Antibody reagents used in this study.

## Data Availability

The datasets generated and analyzed during the current study are available in the NCBI GEO repository, https://www.ncbi.nlm.nih.gov/geo/query/acc.cgi?acc=GSE148416 [79]. The analysis R code generated during the current study is available on GitHub https://github.com/BradRosenbergLab/equinepbmc [80].

## Abbreviations

scRNA-seq: Single-cell RNA sequencing
RNA: Ribonucleic acid
PBMC: Peripheral blood mononuclear cells
CD3: Cluster of differentiation 3
PRF1: Perforin 1
cDC1: Conventional dendritic cell 2
cDC2: Conventional dendritic cell 2
DC: Dendritic cell
T-bet: T-box expressed in T cells
MRSA: Methicillin-resistant *Staphylococcus aureus*
UTR: Untranslated region
ESAT: End Sequence Analysis Toolkit
PCA: Principal component analysis
MHC II: Major histocompatibility complex class 2
CD14: Cluster of differentiation 14
FCGR3A/B: Fc Fragment Of IgG Receptor IIIa
CD16: Cluster of differentiation 16
Ly6C: Lymphocyte antigen 6 C
CD44: Cluster of differentiation 44
SELL: L-selectin
NR4A1: Nerve growth factor IB
CX3CR1: CX3C chemokine receptor 1
HES4: Hes family BHLH transcription Factor 4
CLEC9A: C-type lectin domain family 9 member A
CADM1: Cell adhesion molecule 1
BTLA: B- and T-lymphocyte attenuator
FCER1A: Fc fragment of IgE, high affinity I
SIRPA: Signal regulatory protein a
IRF4: Interferon regulatory factor 4
IRF8: Interferon regulatory factor 8
IRF7: Interferon regulatory factor 7
TCF-4: Transcription factor 4
MS4A1: Membrane spanning 4-domains A1
CD20: Cluster of differentiation 20
CD79A: Cluster of differentiation 79 a
UMI: Unique molecular identifier
ASCs: Antibody-secreting cells
PRDM1: PR domain zinc finger protein 1
BLIMP1: Beta-interferon gene positive regulatory domain I-binding factor
XBP1: X-Box Binding Protein 1
PCNA: Proliferating cell nuclear antigen
TOP2A: DNA topoisomerase 2-alpha
UBE2C: Ubiquitin-conjugating enzyme E2 C
ID3: Inhibitor of DNA binding 3
HIF1A: Hypoxia-inducible factor 1-alpha
MEF2C: Myocyte-specific enhancer factor 2C
TBX21: T-box transcription factor 21
POU2F2: POU Class 2 homeobox 2
Oct-2: Octamer-binding transcription factor 2
IGHD: Immunoglobulin heavy constant delta
IGHM: Immunoglobulin heavy constant Mu
IGHG1: Immunoglobulin heavy constant gamma 1
IGHG3: Immunoglobulin heavy constant gamma 3
IGHG5: Immunoglobulin heavy constant gamma 5
IGHA: Immunoglobulin heavy constant alpha
ZBTB20: Zinc finger and BTB domain containing 20
IGHG6: Immunoglobulin heavy constant gamma 6
ITGAM: Integrin subunit alpha M
ITGAX: Integrin subunit alpha X
CD11b: Cluster of differentiation 11 b
CD11c: Cluster of differentiation 11 c
FCRL4: Fc receptor like 4
FGR: Gardner-Rasheed feline sarcoma viral oncogene homolog
HCK: Hemopoietic cell kinase
AICDA: Activation-induced cytidine deaminase
APEX1: Apurinic/apyrimidinic endodeoxyribonuclease 1
PanIg: Pan immunoglobulin
CD21: Cluster of differentiation 21
CR2: Complement C3d Receptor 2
CD23: Cluster of differentiation 23
FCER2: Fc fragment of IgE receptor II
IgG1: Immunoglobulin gamma 1
IgM: Immunoglobulin Mu
CTSW: Cathepsin W
CD3D: Cluster of differentiation 3 delta
CD3E: Cluster of differentiation 3 epsilon
CD3G: Cluster of differentiation 3 gamma
NK: Natural killer
αβ: Alpha beta
CD8: Cluster of differentiation 8
NKT: Natural killer T
γδ: Gamma delta
GZMK: Granzyme K
GZMM: Granzyme M
GZMA: Granzyme A
CD8A: Cluster of differentiation 8 alpha
CD4: Cluster of differentiation 4
TRDC: T cell receptor delta constant
TRAC: T cell receptor alpha constant
TRBC1: T cell receptor beta constant 1
TRBC2: T cell receptor beta constant 2
GNLY: Granulysin
KLRB: Killer cell lectin like receptor B1
KLRF1: Killer cell lectin like receptor F1
ZNF683: Zinc finger protein 683
HOBIT: Homolog of Blimp-1 in T cells
RT-PCR: Reverse transcription polymerase chain reaction
KLRD1: Killer cell lectin like receptor D1
CD94: Cluster of differentiation 94 (NKG2)
FCER1G: Fc fragment of IgE, high affinity I
CD247: Cluster of differentiation 247
CD2: Cluster of differentiation 2
CD5: Cluster of differentiation 5
MHC I: Major histocompatibility complex class 1
CCR7: C-C chemokine receptor 7
LEF1: Lymphoid enhancer binding factor 1
Th1: T helper 1
Th2: T helper 2
Th17: T helper 17
Flt3: Fms like tyrosine kinase 3
CD13: Cluster of differentiation 13
CD172a: Cluster of differentiation 172a
ANPEP: Alanyl aminopeptidase or aminopeptidase N
BCR: B cell receptor
mRNA: Messenger ribonucleic acid
ISG: Interferon-stimulated gene
