# Supplementary material for: Single-cell resolution landscape of equine peripheral blood mononuclear cells reveals diverse cell types including T-bet+ B cells
Source: BMC Biol. 2021 Jan 22;19:13. doi: 10.1186/s12915-020-00947-5 (PMC7820527; doi:10.1186/s12915-020-00947-5)
Supplement: Supplementary file 1 — Additional file 1: Figure S1. Optimized scRNA-seq data processing workflow improves per cell gene detection. Figure S2. scRNA-seq data processing workflow. Figure S3. Human reference scRNA-seq clustering results and annotation. Figure S4. B cell quality control metrics and antibody secreting cell immunoglobulin isotype usage. Figure S5. T-bet+ B cells identified by scRNA-Seq are detectable by flow cytometry in all subjects examined. Figure S6. Select gene expression patterns in CD3+PRF1+ lymphocyte major cell group. Figure S7. Representative flow cytometry gating schemes for immunophenotyping of equine PBMC. Figure S8. CD3+PRF1− lymphocyte major cell group includes lymphocytes with high expression of ISGs. [file 12915_2020_947_MOESM1_ESM.pdf]

Additional File 1: Supplemental Figures

Single cell resolution landscape of equine peripheral blood mononuclear cells reveals diverse immune cell types including T-bet+ B cells

Roosheel S. Patel, Joy E. Tomlinson, Thomas J. Divers, Gerlinde R. Van de Walle, Brad R. Rosenberg
Corresponding Author: Brad R. Rosenberg E-mail: brad.rosenberg@mssm.edu

Figures S1 to S8

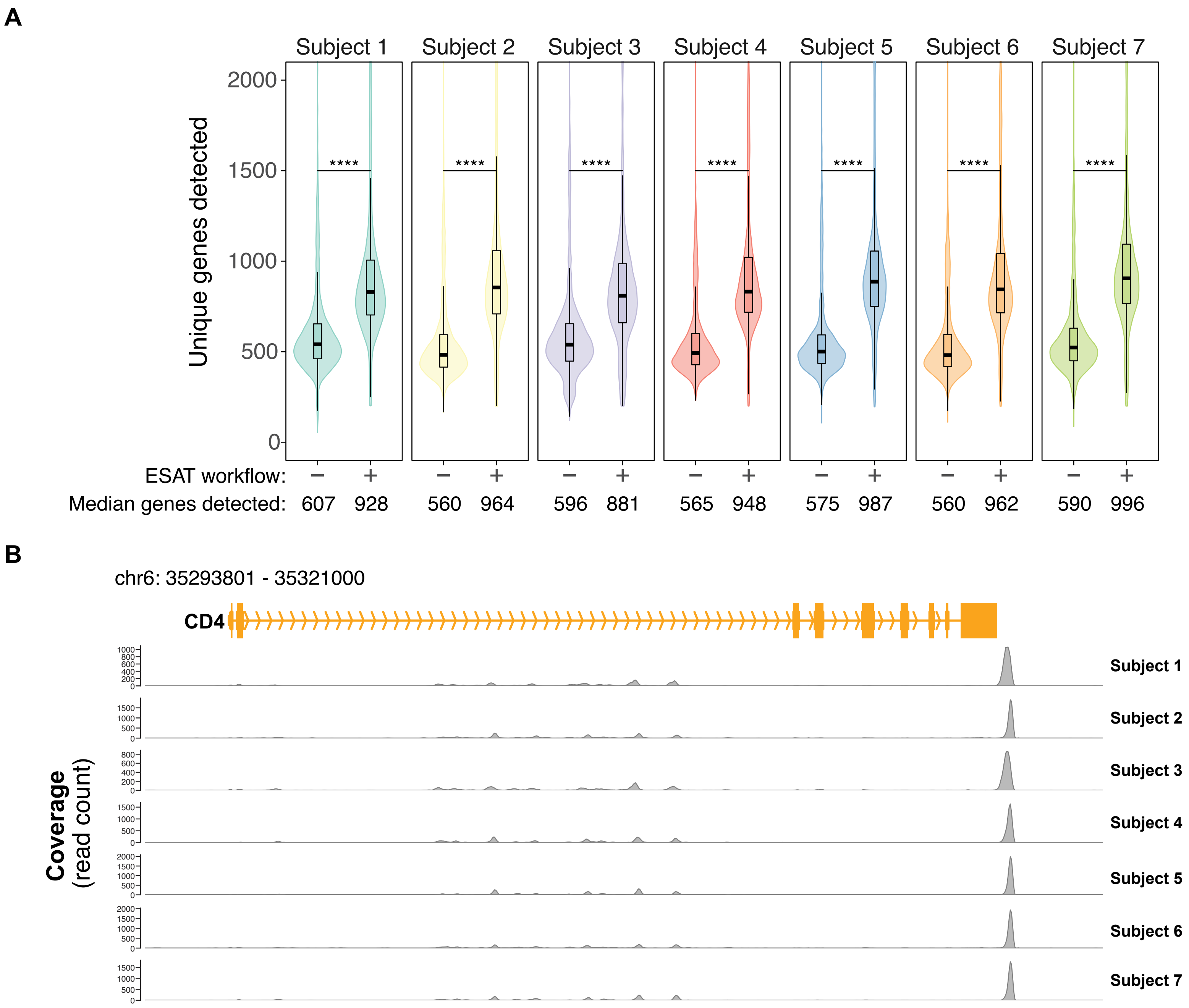

Figure S1. Optimized scRNA-seq data processing workflow improves per cell gene detection. (A) Violin plot of number of unique genes detected per cell across each study subject (N = 7) using standard CellRanger (10X Genomics) workflow versus modified workflow incorporating ESAT. Box plot indicate median, 25th percentile and 75th percentile. Unpaired t-tests were conducted on a per subject basis, **** $p < 1 \times 10^{-15}$. (B) scRNA-Seq read mapping pattern at the CD4 locus, known to be abundantly expressed in equine PBMC, demonstrating majority of reads map directly downstream of reference transcript annotation.

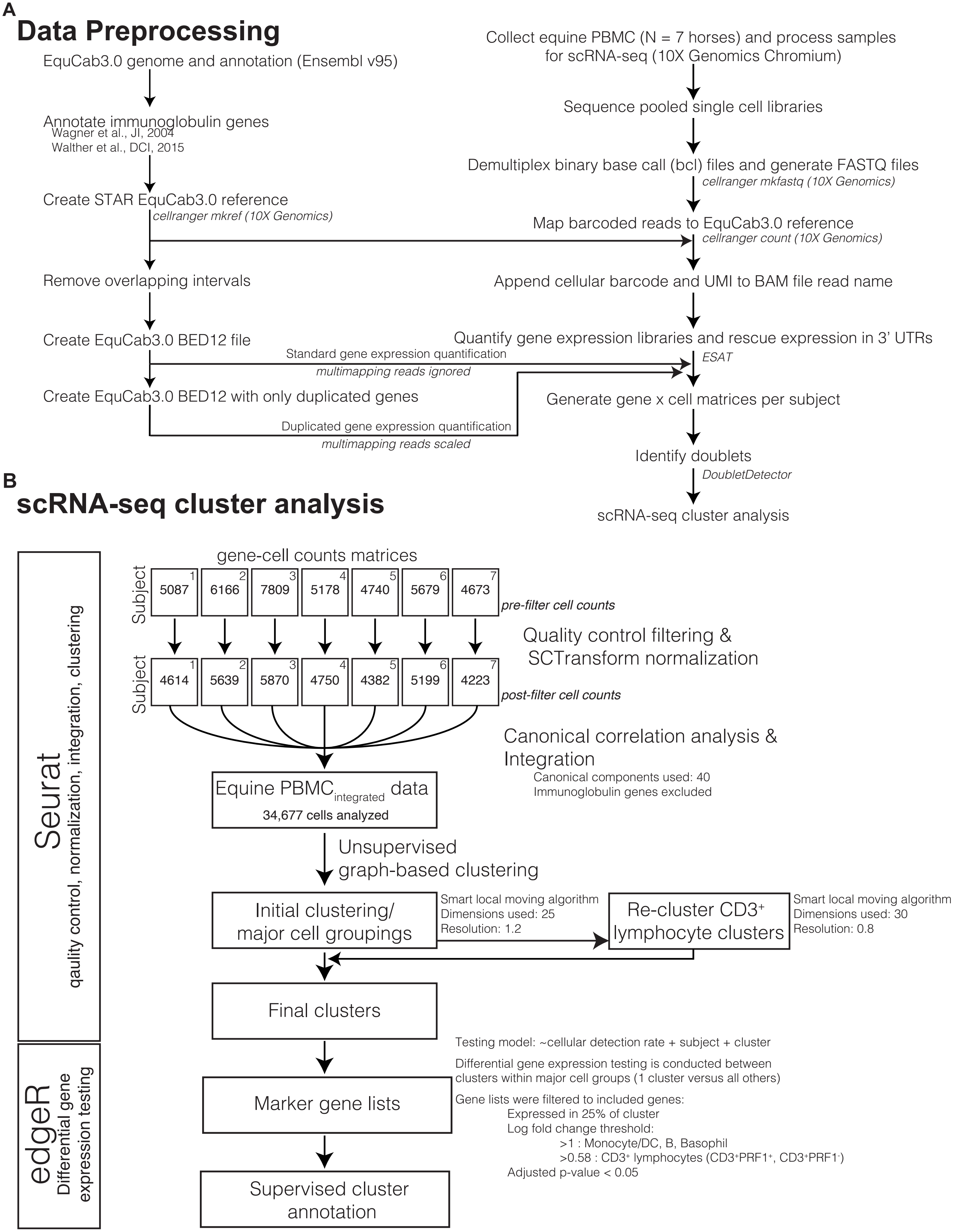

Figure S2. scRNA-seq data processing workflow. (A) Data pre-processing steps from sample collection through gene expression quantification and doublet detection. (B) Data analysis steps for quality control, normalization, integration, clustering and differential gene expression of processed scRNA-seq data for equine PBMCs.

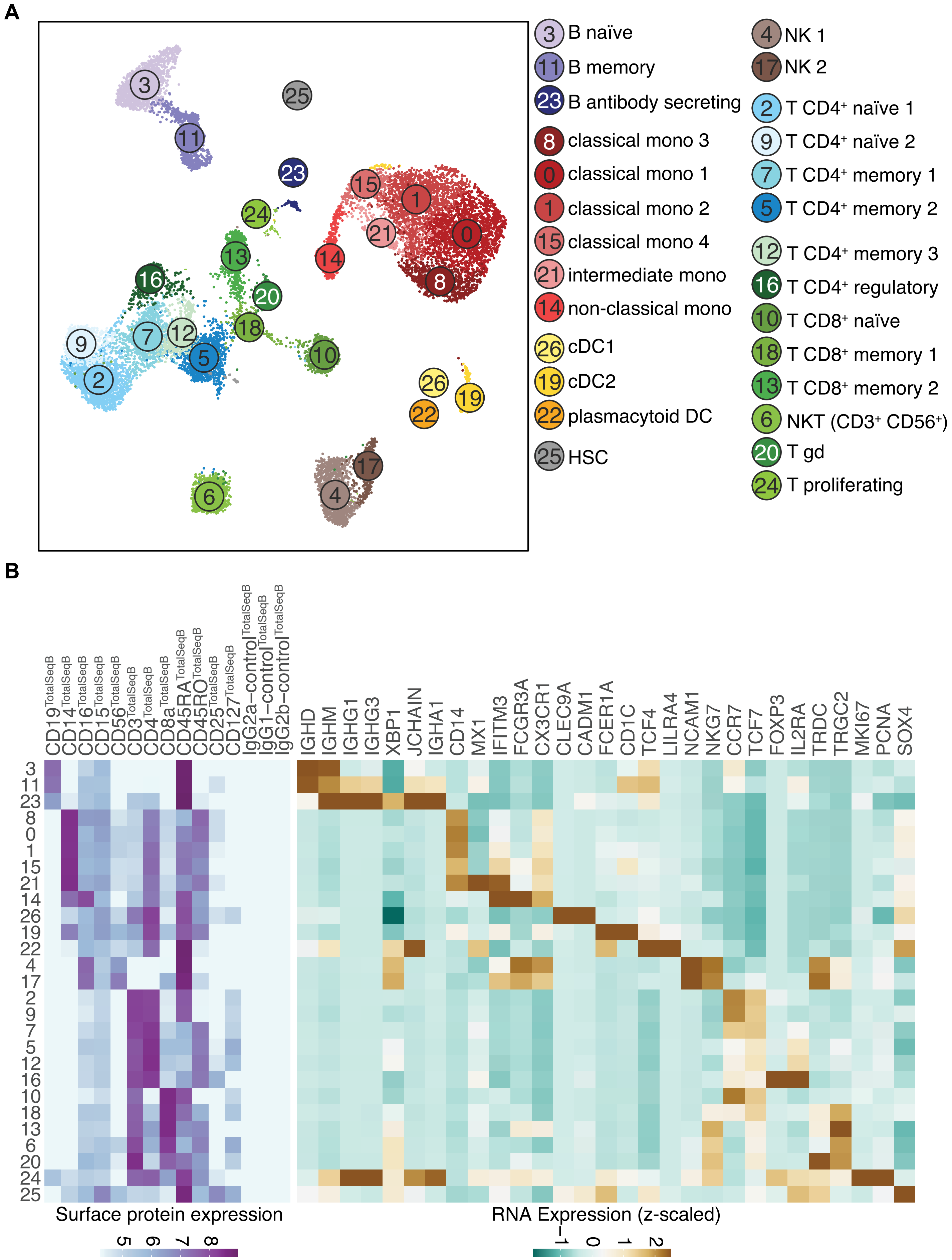

Figure S3. Human reference scRNA-seq clustering results and annotation. Human PBMC scRNA-seq data (surface antibody feature barcoding + gene expression) obtained from the 10X Genomics public dataset collection were integrated and analyzed by Seurat v3. Unsupervised clustering analysis of the integrated dataset identified 26 clusters. (A) UMAP representation of human PBMCs (n = 17,255 total cells from two independent datasets passing QC filters). Points are colored by cluster membership. Clusters were annotated primarily by surface marker labeling when available, supplemented by RNA expression of marker genes. (B) Heatmaps of surface antibody feature barcode data (left) and RNA expression for select marker genes (right) utilized to inform cluster annotation.

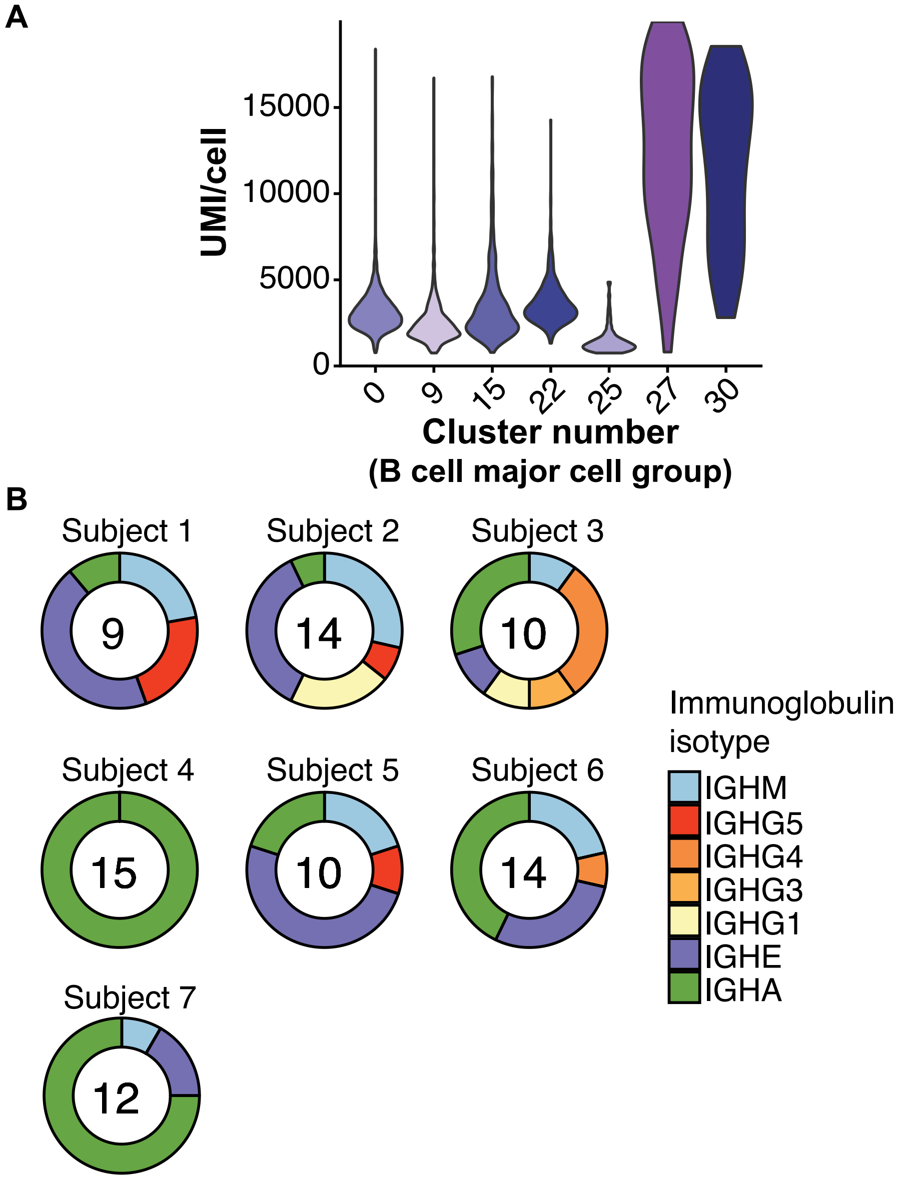

Figure S4. B cell quality control metrics and antibody secreting cell immunoglobulin isotype usage. (A) Violin plot of UMI (transcript) counts per cell across B cell clusters identified by unsupervised clustering. Cluster 25 was excluded from downstream analysis due to insufficient UMI counts. (B) Immunoglobulin isotype usage in antibody secreting cells (cluster 27) as determined by scRNA-Seq gene expression data. Center value indicates number of antibody secreting cells (cluster 27) detected by individual subject.

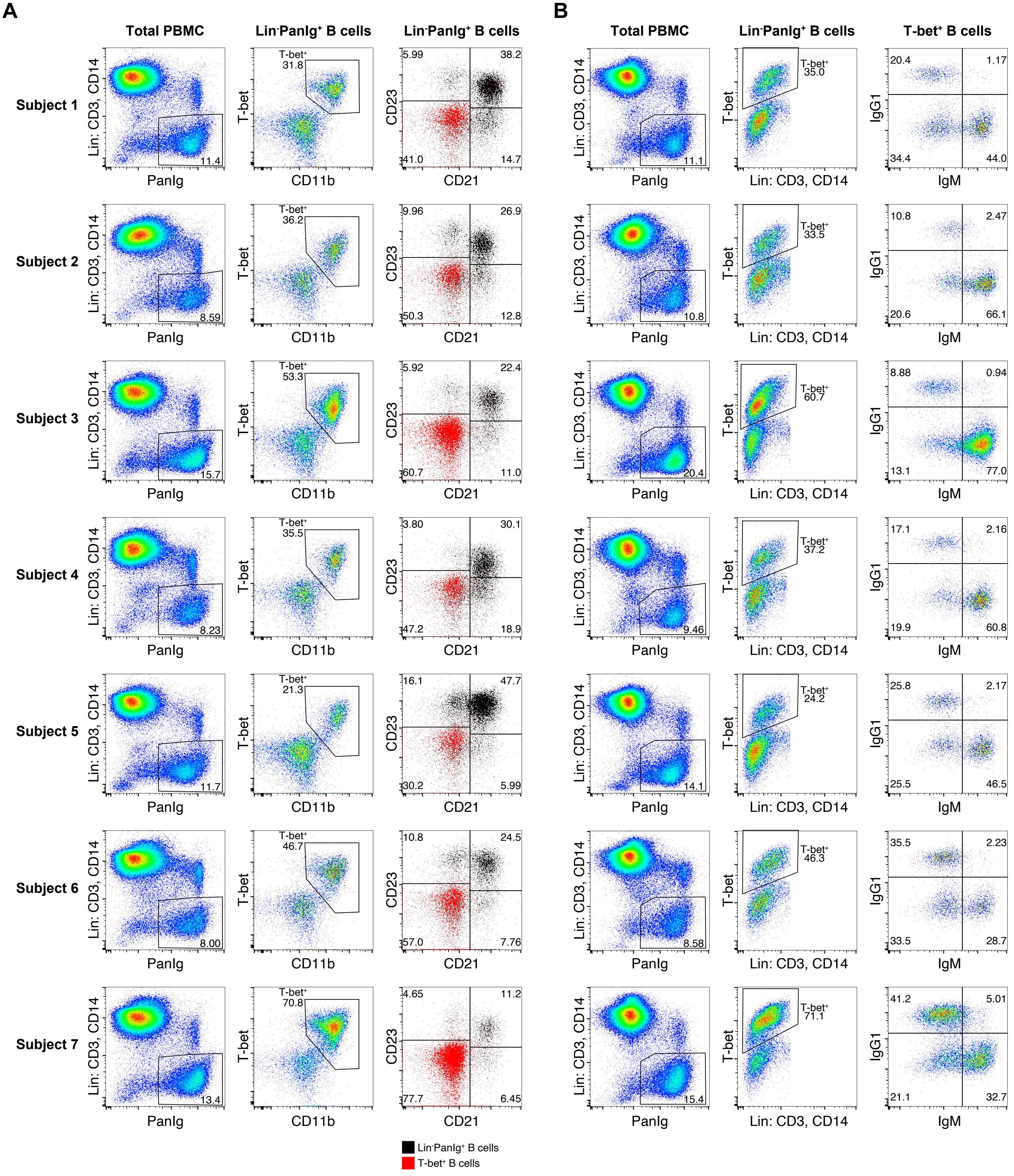

Figure S5. T-bet⁺ B cells identified by scRNA-Seq are detectable by flow cytometry in all subjects examined. Flow cytometry gating schemes for T-bet B cell characterization in equine PBMC across each study subject (N = 7). (A) T-bet⁺ B cell gating for flow cytometry panel including CD11b, CD21 and CD23 labeling. Labels above plots indicate visualized gate. (B) T-bet⁺ B cell gating for flow cytometry panel including IgM and IgG1 surface immunoglobulin labeling. Labels above plots indicate visualized gate.

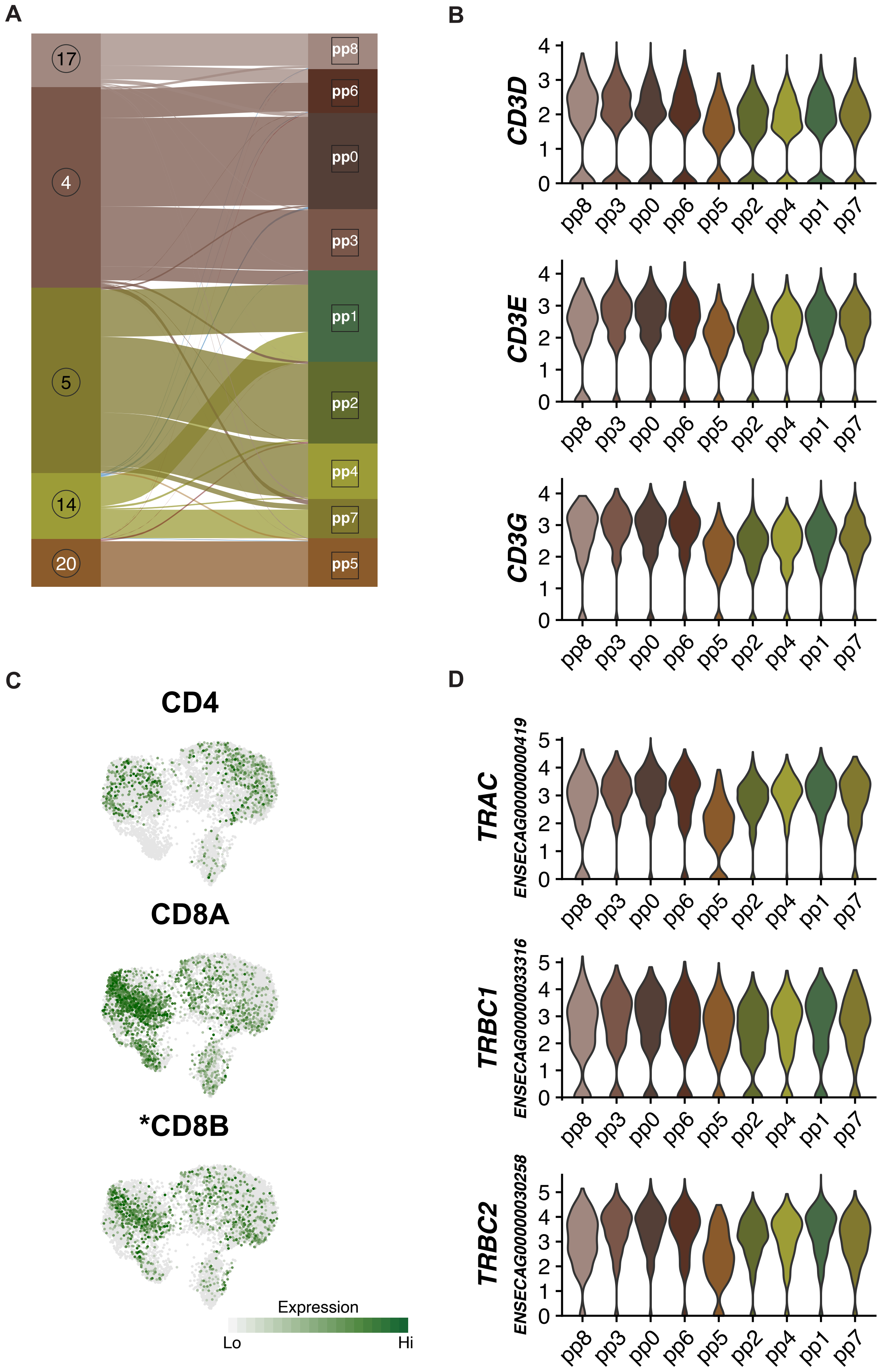

Figure S6. Select gene expression patterns in CD3⁺PRF1⁺ lymphocyte major cell group. (A) Cluster assignments between initial clustering and independent reclustering analyses for CD3⁺PRF1⁺ lymphocyte major cell group. (B) Expression of select CD3 transcripts by indicated clusters in CD3⁺PRF1⁺ lymphocyte major cell group. Values plotted as log normalized counts per cell. (C) Expression patterns of *CD4*, *CD8A* and ENSECAG00000000775 (*CD8B*) in CD3⁺PRF1⁺ lymphocyte clusters. Expression values are scaled independently for each plot, ranging from 2.5 to 97.5 percentile of gene expression across all plotted cells. (D) Expression of T cell receptor genes by indicated clusters in CD3⁺PRF1⁺ lymphocyte major cell group. Values plotted as log normalized counts per cell.

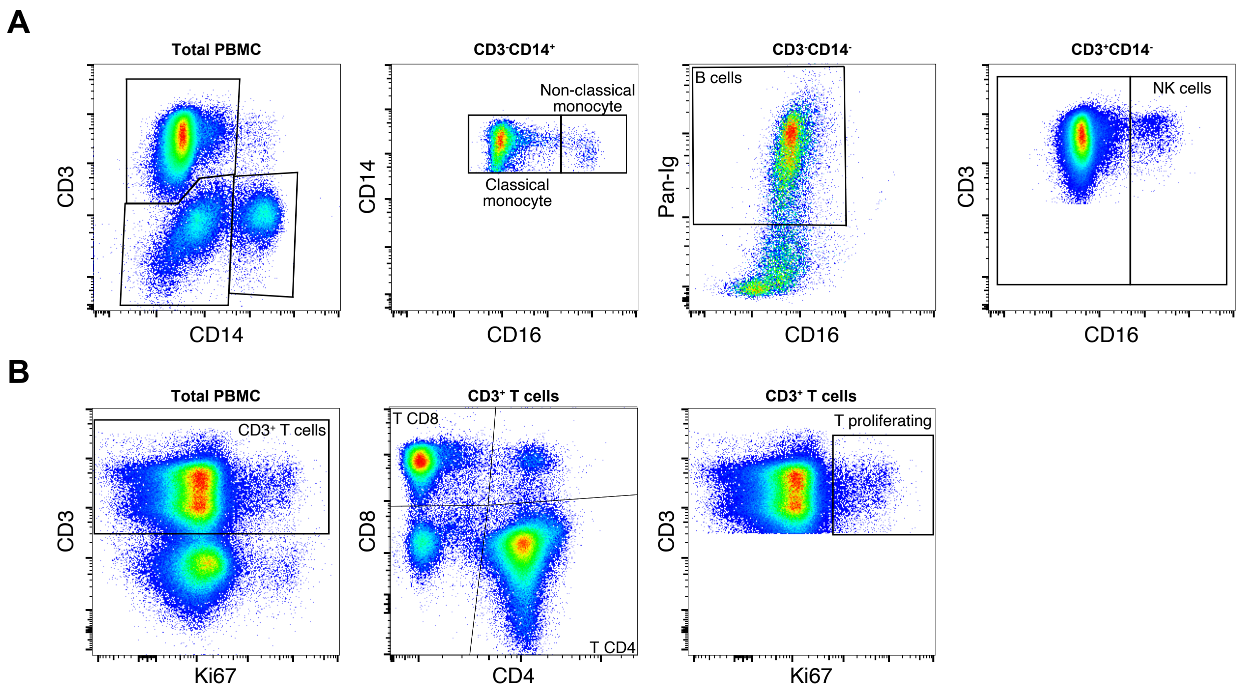

Figure S7. Representative flow cytometry gating schemes for immunophenotyping of equine PBMC. PBMC single cell suspensions were labeled with fluorescent-conjugated antibodies and analyzed by flow cytometry as described in *Materials and Methods*. (A) Gating scheme for immunophenotyping panel to resolve monocyte populations, B cells, and NK cells. Labels above plots indicate visualized gate. (B) Gating scheme for immunophenotyping panel to resolve $CD4^+$, $CD8^+$, and proliferating T cell populations. Labels above plots indicate visualized gate.

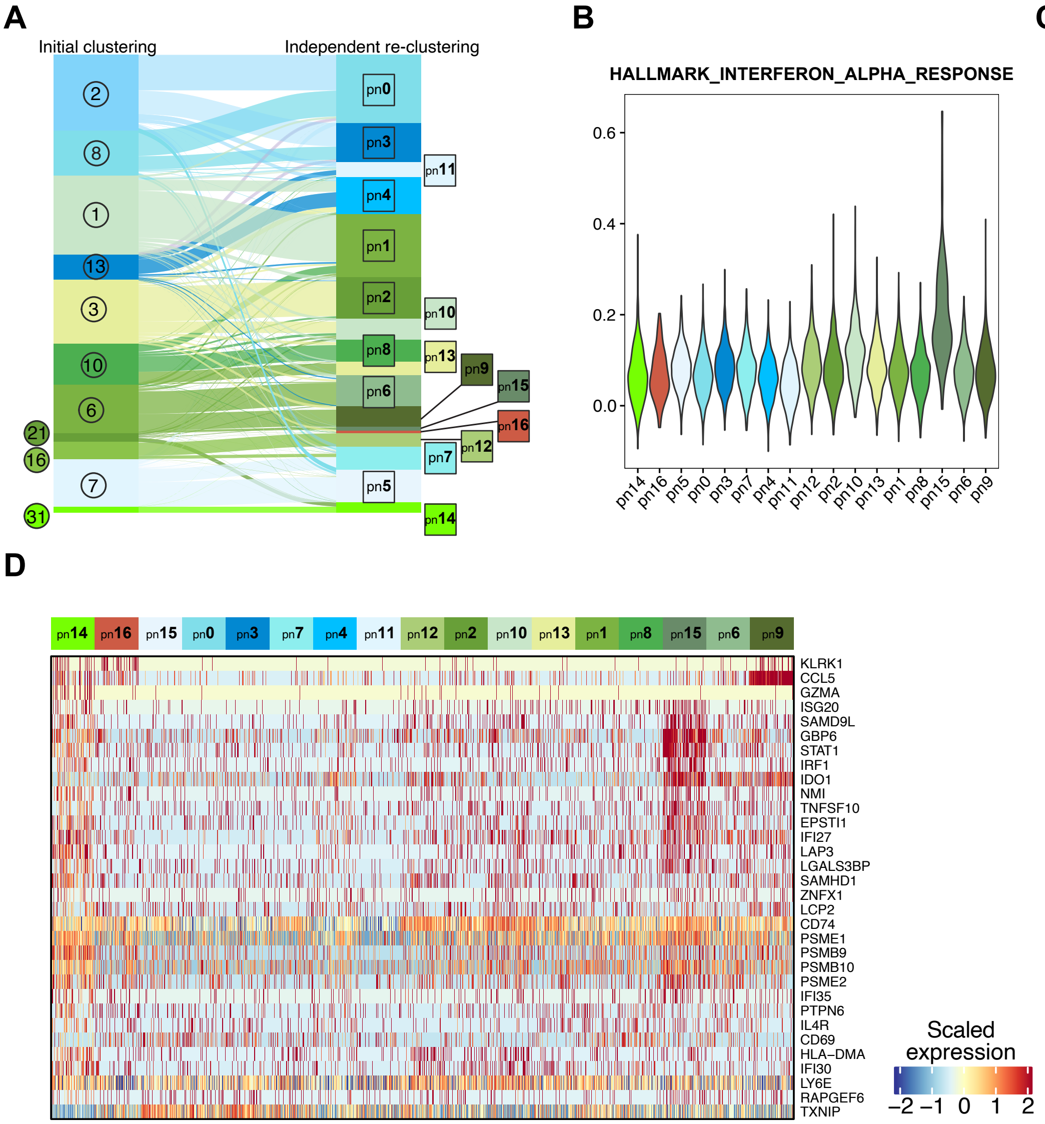

Figure S8. CD3$^+$PRF1$^-$ lymphocyte major cell group includes lymphocytes with high expression of ISGs. (A) Cluster assignments between initial clustering and independent reclustering analyses for CD3$^+$PRF1$^-$ lymphocyte major cell group. (B) Gene expression module scores (calculated per cell with the AddModuleScore function in Seurat) for the Hallmark interferon alpha and (C) Hallmark interferon gamma gene sets from Molecular Signatures Database v7.2 (https://www.gsea-msigdb.org/gsea/msigdb). (D) Heatmap of differentially expressed ISGs (adjusted p-value < 0.05, $\log_2$ fold-change > 0.58 for each cluster versus all other clusters, expressed >25% of cluster, present in hallmark interferon alpha gene set). For each cluster, 100 cells (columns) were randomly selected for plotting purposes.
