# Supplementary material for: Single-cell resolution landscape of equine peripheral blood mononuclear cells reveals diverse cell types including T-bet+ B cells
Source: BMC Biol. 2021 Jan 22;19:13. doi: 10.1186/s12915-020-00947-5 (PMC7820527; doi:10.1186/s12915-020-00947-5)
Supplement: Supplementary file 10 — Additional file 10: Table S1. Antibody reagents used in this study. [file 12915_2020_947_MOESM10_ESM.docx]

**Table S1.** Antibody reagents used in this study

| **Target spp.** | **Antibody** | **Clone** | **Source** | **Reference** |
| --- | --- | --- | --- | --- |
| Eq | CD3-AF647 | UC-F6G | UC Davis, Dr. Stott | [18, 94, 95] |
| Eq | CD4-FITC | CVS4 | Bio Rad Antibodies | [18, 96] |
| Eq | CD8-RPE | CVS8 | Bio Rad Antibodies | [18, 96] |
| Hu | Ki67-PECy7 | B56 | BD Biosciences | This study* |
| Eq | Pan B-cells-RPE | CVS36 | Bio Rad Antibodies | [18, 95] |
| Eq | CD16 | 1A2.D11 | Cornell University, Dr. Antczak | [55] |
| Hu | CD21-BV421 | B-ly4 | BD Biosciences | [18, 97, 98] |
| Eq | CD14-Sav | 105 | Cornell University, Dr. Wagner | [99] |
| Eq | CD14-AF647 | 105 | Cornell University, Dr. Wagner | [99] |
| Eq | CD23-APC-CF750 | 51-3 | Cornell University, Dr. Wagner | [100] |
| Hu | Tbet-PECy7 | 4B10 | BioLegend | This study** |
| Hu | CD11b-PerCP-Vio700 | M1/70.15.11.5 | Miltenyi Biotec | [98] |
| Eq | IgM-CF405M | I-22 | Cornell University, Dr. Wagner | [18, 101] |
| Eq | IgG1-AF488 | CVS45 | Cornell University, Dr. Wagner | [18, 95] |
|  | Live/Dead fixable aqua |  | ThermoFisher Scientific |  |
|  | Live/Dead fixable near IR |  | ThermoFisher Scientific |  |
|  | 7AAD |  | BioLegend |  |

Eq, Horse; Hu, Human

*Anti-human Ki67 clone B56. This clone was validated by test on non-replicating primary equine lymphocytes, which were negative, and pokeweed mitogen stimulated replicating primary equine lymphocytes, which were positive (data not shown).

**Anti-human T-bet clone 4B10 was validated by co-expression patterns with CD11b, CD21, and CD23 as shown in the main manuscript.
